# Brain-wide and cell-specific transcriptomic insights into MRI-derived cortical morphology in macaque monkeys

**DOI:** 10.1038/s41467-023-37246-w

**Published:** 2023-03-17

**Authors:** Tingting Bo, Jie Li, Ganlu Hu, Ge Zhang, Wei Wang, Qian Lv, Shaoling Zhao, Junjie Ma, Meng Qin, Xiaohui Yao, Meiyun Wang, Guang-Zhong Wang, Zheng Wang

**Affiliations:** 1 Department of Endocrine and Metabolic Diseases, Shanghai Institute of Endocrine and Metabolic Diseases, Ruijin Hospital, Shanghai Jiao Tong University School of Medicine, Shanghai, China; 2 Department of Radiology, Ruijin Hospital, Shanghai Jiao Tong University School of Medicine, Shanghai, China; 3 Clinical Neuroscience Center, Ruijin Hospital Luwan Branch, Shanghai Jiao Tong University School of Medicine, Shanghai, China; 4 CAS Key Laboratory of Computational Biology, Shanghai Institute of Nutrition and Health, University of Chinese Academy of Sciences, Chinese Academy of Sciences, Shanghai, China; 5 Shanghai Institute for Advanced Immunochemical Studies, ShanghaiTech University, Shanghai, China; 6 Department of Medical Imaging, Henan Provincial People’s Hospital & the People’s Hospital of Zhengzhou University, No. 7 Weiwu Road, Zhengzhou, Henan China; 7 School of Psychological and Cognitive Sciences; Beijing Key Laboratory of Behavior and Mental Health; IDG/McGovern Institute for Brain Research; Peking-Tsinghua Center for Life Sciences, Peking University, Beijing, China; 8 Institute of Neuroscience, CAS Center for Excellence in Brain Science and Intelligence Technology, State Key Laboratory of Neuroscience, Chinese Academy of Sciences, Shanghai, China; 9 University of Chinese Academy of Sciences, Beijing, China; 10 College of Life Science and Technology, Beijing University of Chemical Technology, Beijing, China; 11 Qingdao Innovation and Development Center, Harbin Engineering University, Qingdao, Shandong China; 12 College of Intelligent Systems Science and Engineering, Harbin Engineering University, Harbin, Heilongjiang China; 13 School of Biomedical Engineering, Hainan University, Haikou, Hainan China

**Keywords:** Genetics of the nervous system, Computational neuroscience

## Abstract

Integrative analyses of transcriptomic and neuroimaging data have generated a wealth of information about biological pathways underlying regional variability in imaging-derived brain phenotypes in humans, but rarely in nonhuman primates due to the lack of a comprehensive anatomically-defined atlas of brain transcriptomics. Here we generate complementary bulk RNA-sequencing dataset of 819 samples from 110 brain regions and single-nucleus RNA-sequencing dataset, and neuroimaging data from 162 cynomolgus macaques, to examine the link between brain-wide gene expression and regional variation in morphometry. We not only observe global/regional expression profiles of macaque brain comparable to human but unravel a dorsolateral-ventromedial gradient of gene assemblies within the primate frontal lobe. Furthermore, we identify a set of 971 protein-coding and 34 non-coding genes consistently associated with cortical thickness, specially enriched for neurons and oligodendrocytes. These data provide a unique resource to investigate nonhuman primate models of human diseases and probe cross-species evolutionary mechanisms.

## Abstract (web summary)

A comprehensive anatomically-defined atlas of brain transcriptomics in macaques is still lacking. Here, the authors generate complementary bulk RNA-seq and snRNA-seq datasets from cynomolgus macaques to examine the link between brain-wide gene expression and regional variation in morphometry.

## Introduction

Large-scale magnetic resonance imaging (MRI) and genetics datasets have opened up entirely new avenues to discover common genetic variants contributing to MRI-derived structural and functional phenotypes of the human brain in health and disease^1–5^. Given the functional heterogeneity of the brain, transcriptomic-based analyses have been conducted across the entire brain utilizing high-throughput profiling of tissues, cells, and most recently at the level of single nucleus in order to gain unprecedented insights into the neurobiological mechanisms by which genetic and molecular differences influence cognition, behavior and emotion in different species^6–9^. A resource portfolio like the Allen Brain Atlas containing a multimodal atlas of gene expression and anatomy from the prenatal period to adulthood is essential to enable advanced data-mining for researchers interested in comparisons across brain organization or development^8,10,11^. Cumulative evidences reveal a hierarchical gradient pattern of cortical microstructure, functional connectivity, and gene expression profiling in the primates neocortex mainly spanning from primary sensorimotor regions to transmodal regions^12–14^. However, such brain-wide observations fail to capture the intrinsic complexity within specific brain regions due to relatively sparse spatial sampling coverage that restricts the types of analyses^8,15,16^. Therefore, up to date, cross-species transcriptomic comparisons have primarily relied on data from a small set of brain areas in the monkey brain^17,18^, which apparently restrains the capacity to examine relationships between genes with spatial profiles of regional expression and whole-brain imaging-derived phenotypes. Furthermore, a complete anatomically defined atlas of brain transcriptomics in nonhuman primates, although still lacking^7,19,20^, is prerequisite to bridge the gap between microscale attributes including a single-cell/nucleus transcriptomic atlas^6^ and a wide variety of macroscale imaging attributes that inform brain development, morphology and function^16,21–23^.

It has long been recognized that characterization of spatial and temporal associations between gene expression and brain structural variation is a critical step towards developing a mechanistic model of how specific genes influence brain infrastructure. In humans, genome-wide association meta-analyses of brain MRI data from multiple large-scale repositories have demonstrated that distinct genes among the highly polygenic architecture of the human cerebral cortex contribute to the development of specific cortical areas^2,3,24–26^, which engenders brain structural variations and causally linked differences in brain functional specializations. Using coarse partitions of the human cortex, previous studies have identified polygenic organization patterns related to variation in global and regional cortical thickness (CT) during development^27–29^, which may confer the potential to detect regional vulnerability to pathological changes at the earliest stages of neuropsychiatric disorders^4,30–35^. Notably, as a simple pragmatic surrogate characterizing cortical morphometry, average thickness has been attributed to an array of complex biological processes including intracortical myelination, remodeling of dendritic arbors, axonal sprouting and its components (e.g., neuronal and glial cells, neuropil and dendritic spines)^32,36,37^, whereby transcriptomic divergence observed between human and macaque brains is likely to be anatomically dependent within the brain^17^. However, little is known in nonhuman primates about how common genetic determinants mediate the thickness of the cerebral cortical sheet across the entire brain, despite prior reports of developmental variations in human^29^ and monkey brains^7^. Furthermore, it remains unclear whether biological and cell pathways that underlie regional variability in CT differ between monkeys and humans.

In this study, we generated complementary atlas-based bulk-tissue RNA-sequencing (RNA-seq) dataset of 878 samples from 111 regions defined by the D99 template of macaque brain^38,39^ and single-nucleus RNA-sequencing (snRNA-seq) data, and structural MRI data acquired from 162 cynomolgus macaques^40^. Firstly, we evaluated brain-wide structural variation and composition of differentially expressed genes, and depicted the cortical landscapes of various neurotransmitter-related genes. We placed an emphasis on transcriptional heterogeneity within the frontal lobe to characterize the frontal-specific genetic architecture using weighted gene co-expression network analysis (WGCNA). Second, using this anatomically defined gene expression and structural MRI datasets, we applied partial least squares (PLS) regression to test consensus molecular correlations with MRI-derived CT. Moreover, we used both the publicly accessible and our single-nucleus sequencing data to resolve the underlying diversified cell types enriched in those CT-related genes. Third, we performed a functional enrichment analysis to infer ontological pathways, which converge with myelin, spine, dendrite and neuron-projection terms. Finally, we linked clusters of CT-related genes to cell types, specifying neurons and oligodendrocytes as contributing most to the transcriptomic relationship of cortical structural variations. Together, we provide a comprehensive spatial transcriptional atlas of macaque brain which allows us to integrate microscale single-cell/nucleus RNA-seq and macroscale brain imaging for elucidating the brain-wide molecular and genetic architecture of brain phenotypes.

## Results

### Characterization of brain-wide transcriptome

**Fig. 1:**
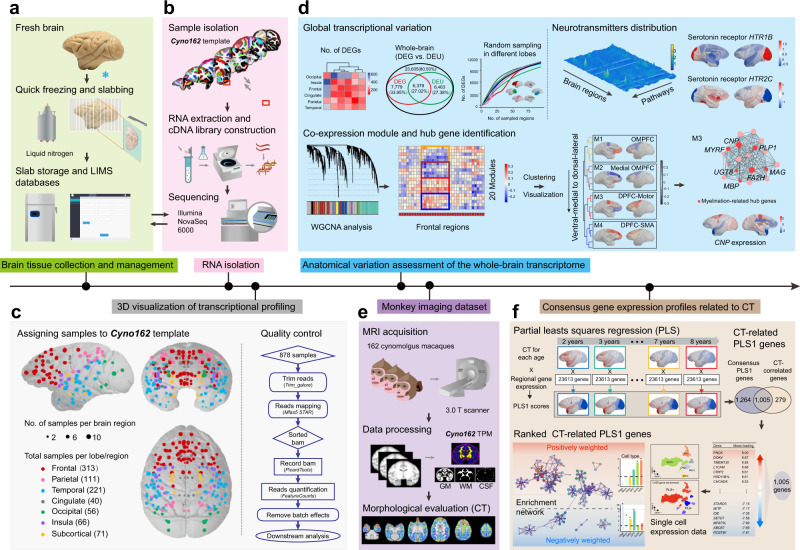
A scheme of experimental strategy, data generation and analysis pipeline. **a** Brain tissue collection and management. The fresh brain of cynomolgus macaques was immediately frozen in liquid nitrogen, and coronally cut into a serial of slabs (2-mm thickness). Each slab was placed on a barcoded disk, photographed for orientation, stored at −80 °C and documented by an in-house developed LIMS (Laboratory Information Management Systems) software. **b** RNA isolation. For each slab, both digital photographs (taken before and after sampling) and corresponding MRI images from an atlas of cynomolgus macaques *Cyno162* were used to mark the exact spatial location of the individual sampled area. The sampled tissues were hence subject to lysis, standardized RNA extraction and cDNA library construction, and finally sequenced using a high-capacity Illumina NovaSeq 6000 instrument. **c** 3D visualization of all collected samples based on the *Cyno162* brain template. The size of each color-coded sphere indicates the number of samples collected from that specific brain region. The total numbers of samples collected from each brain lobe/region are shown here. A total of 878 samples were subject to quality control (see “Methods” for details) and then to subsequent transcription profiling. **d** Anatomical variation assessment of the whole-brain transcriptome. DEG, DEU and WGCNA analyses were performed to explore global transcriptional variation, and to identify both co-expression modules and region-specific hub genes (upper left and lower panels). Brain-wide topography of transcript distributions for neurotransmitters was plotted in 3D space and rendered onto the brain surface (upper right panel). DEG, differential expressed gene; DEU, differential exon usage; WGCNA, weighted gene co-expression network analysis. **e** Monkey neuroimaging dataset, data processing and morphological evaluation including estimation of cortical thickness (CT). **f** Consensus gene expression profiles related to CT. Partial least squares (PLS) regression analyses were applied to identify candidate gene clusters whose expression were associated with the spatial variation of CT over the whole brain at different ages. Cellular diversity of CT-related genes was assessed with single-cell expression data and functional annotations were evaluated.

**Fig. 2:**
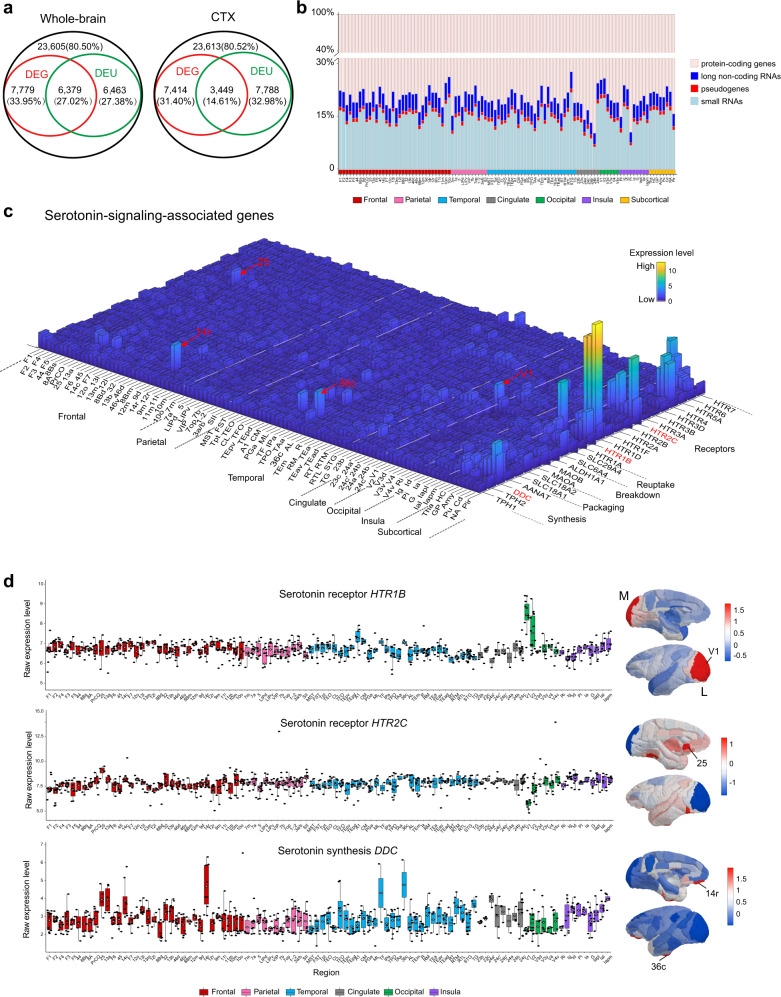
Transcriptional composition and topography of expression distributions for serotonin-signaling-associated genes. **a** Venn diagrams demonstrate the total number of expressed genes among 29,324 annotated genes and the overlaps between spatially DEGs and DEUs for whole-brain (left) and cortex (right). Percentages of DEGs and DEUs were calculated by dividing the corresponding expressed genes. DEGs, differentially expressed genes; DEUs, differential exon usage genes. **b** Percentages of different types of DEGs detected in 102 regions that were sampled thrice or more, including protein-coding genes, long non-coding RNAs, pseudogenes, and small RNAs, scaled to the maximum height for each brain region. **c** Expression topography of serotonin-signaling-related genes across the entire brain. Expression profiles for individual genes were transformed to the same scale by normalizing to the median expression value and then plotted in exponential form. Highly expressed genes are marked in corresponding brain structures. **d** Box plots of raw expression levels of three representative genes associated with serotonin (*HTR1B*, *HTR2C* and *DDC*) at each of the 97 neocortical brain regions and the normalized spatial expression patterns on the cortical surface are displayed alongside. In each box plot, the center line indicates the median, the edges of the box indicate the 25th and 75th percentile (interquartile range, IQR) and the whiskers indicate last point within a 1.5× IQR (sample size varies among brain regions, ranging from 2 to 15, see Source data). All sampled areas are color coded by major structure; the bar below from left to right are frontal, parietal, temporal, cingulate, occipital and insula. M, medial view; L, lateral view. See Supplementary Data 1 for a complete list of abbreviations for all sampled regions. Source data are provided as a Source Data file.

This study combined single-nucleus, bulk-tissue transcriptomics, and structural MRI data to determine links between cell-typespecific gene expression and variations in cortical morphometric features in macaque monkeys, and established a multimodal data generation and analysis pipeline for imaging transcriptomics as illustrated in Fig. 1. To create a brain-wide, anatomically defined, spatial transcriptome atlas of the macaque brain, we profiled genome-wide expression from 819 samples (RNA integrity number, 8.20 ± 0.68, mean ± s.d.) originating from 110 brain regions by total RNA-seq, pooling across both left and right hemispheres after strict quality control (Fig. 1 and Supplementary Data 1). This monkey atlas manifested as relatively dense spatial sampling of the frontal and temporal lobes compared to the spatial distribution of tissue samples in the Allen Human Brain Atlas (Supplementary Fig. 1). The deep sequencing produced an average of 52.7 million reads per sample, which enabled the detection of protein-coding and non-coding genes, as well as accurate estimation of lowly expressed genes. Only genes with detected reads in ≥10% samples were considered as ‘expressed’. Consequently, 80.50% (23,605 out of 29,324 annotated genes) of the genes were detected with expression signal across the macaque brain (Fig. 2a and Supplementary Data 2). A similar proportion of expressed genes (80.52%, 23,613 genes) were detected when only the cortical regions were considered.

To identify genes with conserved expression patterning across brain regions, we performed differential expression gene (DEG) and differential exon usage gene (DEU) analysis for every pair of 102 anatomically defined brain structures (94 cortical and 8 subcortical areas) that were sampled thrice or more^41^. Our result revealed that 59.98% of expressed genes were DEGs between any two regions, and 46.00% were DEGs between any two cortical areas. By contrast, 54.40% of expressed genes were DEUs between any two regions, and 47.59% were DEUs across cortical areas. Moreover, 27.02% and 14.61% of the expressed genes were detected as DEGs and DEUs, respectively, across the entire brain and the cortex-only areas. In the meantime, 33.95% and 27.38% of the expressed genes were restrictedly detected as either DEGs or DEUs between any two regions, while 31.40% and 32.98% expressed genes were detected as DEGs or DEUs between cortical areas. These results demonstrate widespread spatial variations in the expression of entire transcripts or individual exons, suggesting that alternative splicing assessment is of equivalent importance as DEGs for assessing regional gene expressions. Moreover, transcriptome-wide analysis of the present dataset allowed us to identify multiple compositions of DEGs that include highly expressed protein-coding genes (79.73 ± 3.10%, mean ± s.d.), long non-coding RNAs (4.48 ± 0.46%), small RNAs (14.77 ± 2.79%) and pseudogenes (1.01 ± 0.12%) (Fig. 2b).

In addition, this transcriptomics atlas enables us to generate a bird’s eye view of gene expression patterns related to specific neurotransmissions across the entire brain, for instance, showing a spatially-resolved enrichment of serotonin-signaling-associated genes (Fig. 2c). Serotonin is well known to form the most complex efferent system in the primate neocortex and plays a crucial role in the control of complex brain functions and neuropsychiatric disorders^42–45^. Representative regional enrichments relevant to serotonin synthesis, packaging, breakdown, reuptake, and receptors were rendered onto a 3D brain surface map for demonstration (Fig. 2d). We found that the *serotonin receptor 1B* (*HTR1B*), *serotonin receptor 2C* (*HTR2C*) and serotonin synthesis-related gene *L-Dopa-Decarboxylase* (*DDC*) were highly enriched in the primary visual cortex (V1), medial prefrontal area (area 25), and subdivision of orbitomedial prefrontal cortex (area 14r) and caudal subdivision of perirhinal cortex (area 36c), respectively. The expression landscapes of these transmitter genes closely resembled the corresponding mRNA expression profiles in the human brain^46^ (Supplementary Fig. 2). Interestingly, the overall expression pattern of *HTR1B* was consistent with the PET tracer image obtained from human subjects^47^. We provided 3D plots of transcript distributions across the whole brain and brain maps for other neurotransmitter systems in Supplementary Fig. 3.

### Regional transcriptional signatures in macaque brain

**Fig. 3:**
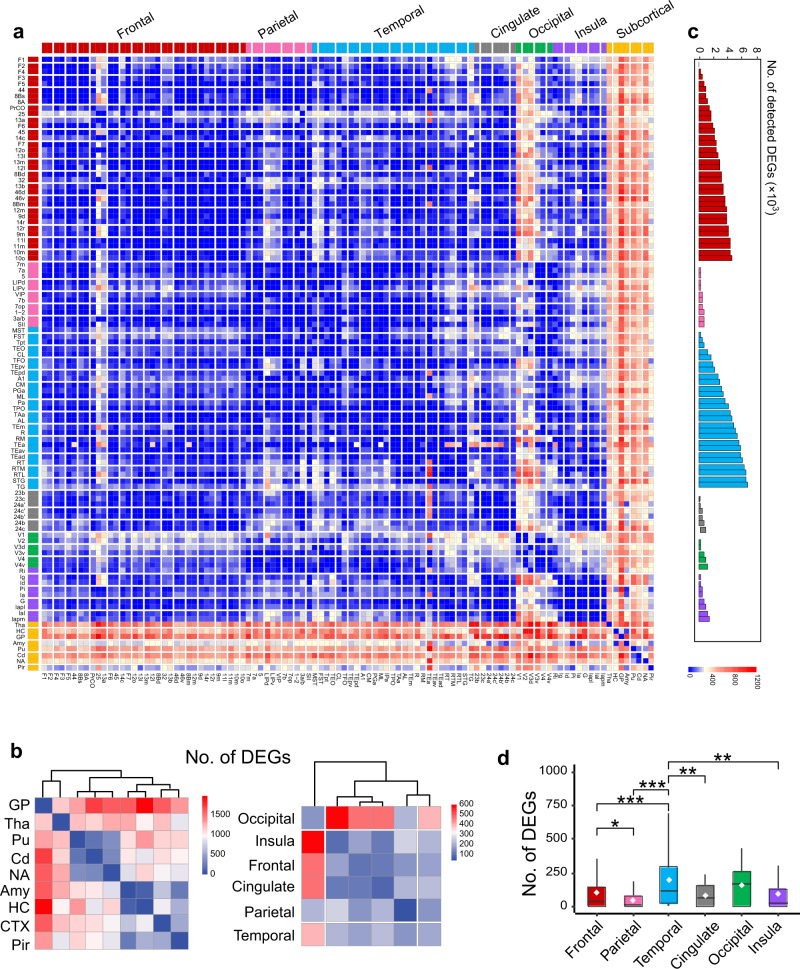
Brain-wide structural variation in gene expression in macaques. **a** Pairwise differential expression matrix across the brain. Each matrix entry represents the upregulated (bottom left) and downregulated (top right) number of DEGs with a fold change ≥ 2 in expression level. Subcortical and occipital regions exhibit marked heterogeneous patterns (red) in contrast to the rest cortical regions (blue). DEGs, differentially expressed genes. **b** Heatmap of the numbers of DEGs between major brain structures (left) and cortical regions (right). Occipital regions exhibit marked inter-regional variations (red). GP globus pallidus, Tha thalamus, Pu putamen, Cd caudate, NA nucleus accumbens, Amy amygdala, HC hippocampus, CTX cortex, Pir piriform cortex. **c** Increased number of detected DEGs when increased the number of randomly-sampled areas within each lobe/region (color coded) (Permutation test, *n* = 1000). **d** Average number of DEGs in each lobe/region. In each box plot, the center line indicates the median, the white diamonds indicate the mean, the edges of the box indicate the 25th and 75th percentile (interquartile range, IQR) and the whiskers indicate last point within a 1.5× IQR (*n* = 561, frontal; 55, parietal; 351, temporal; 21, cingulate; 15, occipital; 36, insula). *p < 0.05, **p < 0.01, ***p < 0.001, pairwise two-sided Wilcoxon rank sum test (uncorrected for multiple comparisons)*, p* values in **d** are provided as a Source Data. Source data are provided as a Source Data file.

To capture the fundamental architecture of the macaque brain transcriptome, we plotted a pairwise differential expression matrix which shows upregulated (bottom left) and downregulated (top right) DEGs (Fig. 3a), further validated by applying DEU analysis and other DEGs detection methods including edgeR and limma (Supplementary Fig. 4). Heterogeneity of differential gene expression between divisions of the macaque brain is prominent, manifested as distinctively regulated patterning of subcortical and occipital cortices relative to the rest of cerebral cortex (Fig. 3b), compatible with that in human brain^48^ and supported by principal component analyses (Supplementary Fig. 5). Remarkably, we were able to detect more DEGs especially in the frontal and temporal lobes (Fig. 3c) and observe nonnegligible differences even within the frontal lobe. More DEGs were detected in the temporal lobe while the average number of DEGs in the frontal lobe was comparable to that of other cortical lobes/regions (Fig. 3d).

### Genetic topography in macaque brain

**Fig. 4:**
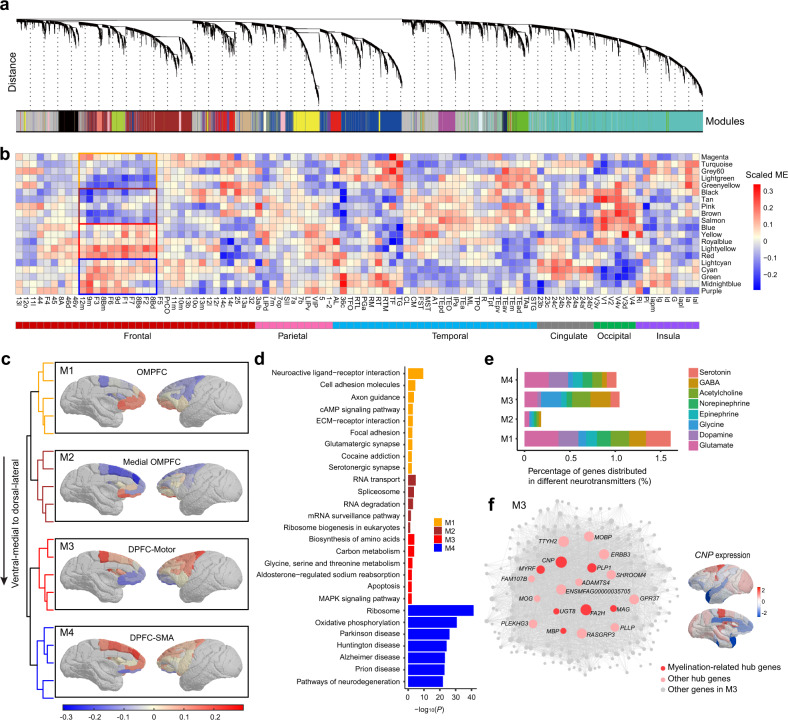
Global and frontal-specific co-expression modules in macaque brains. **a** WGCNA gene hierarchical clustering dendrogram and modules of co-expression. Y-axis represents the co-expression distance between genes while x-axis represents individual gene. A total of 20 distinct co-expression modules are identified, visualized by colored bars at the bottom of the dendrogram. **b** Module-region association. Heatmap plot of hierarchical clustering of these 20 modules with anatomic ordering from the frontal lobe to the insula. Each module is grouped by specific gene clusters. ME module eigengene. **c** The module dendrogram and anatomical patterning of four modules (M1–M4) identified in (**b)** with averaged eigengenes in frontal regions are demonstrated on cortical surface. OMPFC orbitomedial prefrontal cortex, DPFC dorsal prefrontal cortex, SMA supplementary motor area. **d** Top functional enrichments for genes identified in M1, M2, M3 and M4, which were sorted by FDR-corrected −log_10_(*P*). Statistical significance of the overlaps between each gene set and each module was assessed by a one-sided hypergeometric test and FDR corrected. **e** Percentage of marker genes enriched in different neurotransmitters in M1–M4. **f** Illustration of gene–gene connections for module M3, including top 19 hub genes. For visualization, 185 genes with intra-module expression similarity >0.3 were shown. Myelination-related hub genes, like *CNP*, are highly expressed in some cortical regions. Source data are provided as a Source Data file.

We performed WGCNA analysis based on 19,971 genes that were expressed solely in the cortical regions following the standard procedures^49,50^. Genes were determined by summing up all transcripts including protein-coding genes (PCGs), long non-coding RNAs (lncRNAs) and microRNAs (except mitochondrial RNAs), and the top 95% of highly expressed genes were retained for analysis to reduce the noisy effect of low-expression genes (Fig. 4a). A total of 20 main transcriptional modules were derived, each of which was represented by a characteristic gene cluster across 97 cortical regions. The expression patterns summarized by module eigengene in each region were plotted (Fig. 4b, Supplementary Figs. 6a, 7, and Supplementary Data 3). Given the heterogeneity and complexity of the primate prefrontal cortex, we were particularly interested in exploring its spatial organization pattern of gene expression. With hierarchical clustering analysis, four modules (M1–M4) of gene expression were identified and spatially organized along a dorsolateral-ventromedial gradient within the frontal lobe, where M1 and M2 modules mainly spanned the medial section while M3 and M4 modules pertained to the dorsal section (Fig. 4c and Supplementary Fig. 6b).

We further conducted Kyoto Encyclopedia of Genes and Genomes (KEGG) pathway enrichment analyses on genes assigned to M1–M4 (Fig. 4d and Supplementary Data 4). Interestingly, module M1 was enriched in neuronal structure and transmitter related annotations, including neuroactive ligand-receptor interaction (*p* = 3.35 × 10^−10^, FDR corrected for all comparisons below), cell adhesion molecules (*p* = 3.25 × 10^−5^), axon guidance (*p* = 2.17 × 10^−4^), glutamatergic synapse (*p* = 2.56 × 10^−3^), and serotonergic synapse (*p* = 4.92 × 10^−3^). M2 was uniquely associated with multiple biological processes related to RNA synthesis and degradation, such as RNA transport (*p* = 1.83 × 10^-5^) and RNA degradation (*p* = 9.18 × 10^−4^). In contrast, M3 showed close associations with biosynthesis of amino acids (*p* = 1.29 × 10^-4^), carbon metabolism (*p* = 1.76 × 10^−4^), glycine, serine, and threonine metabolism (*p* = 1.94 × 10^−3^). M4 was highly enriched in supplementary motor area and dorsal prefrontal cortex, of which the genes were associated with neurodegenerative process and diseases, such as ribosome (*p* = 5.25 × 10^−42^), oxidative phosphorylation (*p* = 2.01 × 10^−31^), Parkinson disease (*p* = 7.54 × 10^−27^), Huntington disease (*p* = 5.15 × 10^−25^), and Alzheimer disease (*p* = 6.73 × 10^−24^).

Using previously published gene lists^8^, we evaluated the spatial distribution of different neurotransmitters in these modules. It showed that serotonin (odds ratio, OR = 4.78, *p* = 0.001, FDR corrected for all comparisons below, Fisher’s exact test), dopamine (OR = 5.17, *p* = 0.001) and glutamate (OR = 3.29, *p* = 0.001) related genes were enriched in M1, whereas glycine genes were enriched in M3 (OR = 4.75, *p* = 0.009) (Fig. 4e and Supplementary Data 5). The dorsolateral-ventromedial gradient of gene ensembles within the frontal lobe was observed similar to the expression pattern of those neurotransmitters-related genes (Supplementary Fig. 8). The brain-wide and frontal-specific gene expression profiles were demonstrated in Supplementary Data 6. We then visualized the network of M1–M4 modules and identified the hub genes with high degree (degree > 100) and significant Pearson correlation with the corresponding module eigengene (*k*_ME_, *p* < 0.05)^51,52^. Of the 19 hub genes in M3, 7 of them were myelination-related genes (*CNP*, *FA2H*, *PLP1*, *MYRF*, *UGT8*, *MAG*, *MBP*) (Fig. 4f and Supplementary Data 7). Notably, myelination-related genes, like *CNP*, were highly expressed in cortical regions, consistent with the notion that primary cortical areas are strongly myelinated in both human and macaque^29,53^. Detailed hub gene diagrams of other three modules and cell type enrichment for all modules annotated with three independent snRNA-seq datasets are provided in Supplementary Fig. 9, 10 and Supplementary Data 7, 8.

### Persistent gene expression profiles related to brain-wide variations in cortical thickness

**Fig. 5:**
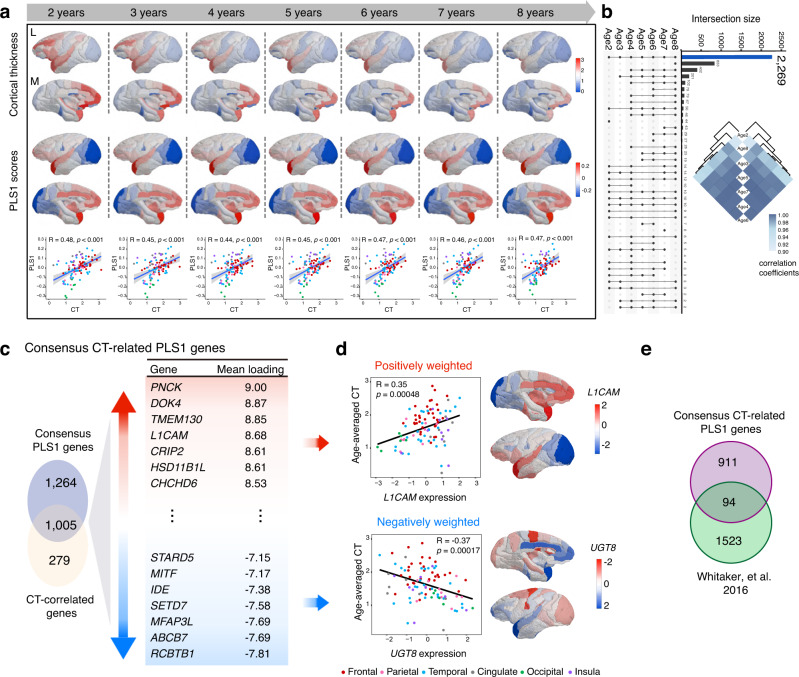
Consensus gene expression profiles related to dynamic variation of regional cortical thickness. **a** Cortical maps of regional CT at different ages, and corresponding weighted gene expression maps of regional PLS1 scores (weighted sum of 23,613 gene expression scores). The bottom panel shows scatterplots of regional CT (x-axis) versus regional PLS1 scores (y-axis) that are associated with a list of genes with rank denoted by the correlation between the first PLS component and respective gene expression maps. The blue line is the fitted line and gray shaded area indicates the 95% confidence intervals. *p* values are provided as a Source Data. CT, cortical thickness; PLS1, the first partial least squares component; L, lateral view; M, medial view. **b** UpSet plot showing intersections of PLS1 genes with normalized weights Z score > 3 or < −3 from age 2 to 8. The bar encoding the size of genes intersecting in all age groups is colored in blue. The similarity matrix shows the relationship between PLS1 scores of intersecting genes between different age groups. **c** Consensus CT-related PLS1 genes and corresponding functional annotation. Left: Venn diagram showing the overlap between identified PLS1 genes (2269 genes) and CT-correlated genes (1284 genes). Right: Genes that are strongly positively weighted on PLS1 are in red, while those negatively weighted ones are in blue. Loadings reflect the shared variance between the original variables and the PLS scores, which represents the degree of contribution of each variable to the latent variable identified by PLS. **d** Scatterplots of normalized expression of typical positively/negatively weighted PLS1 genes (e.g., *L1CAM* and *UGT8*, x-axis) versus averaged regional CT (y-axis) over major structures. Cortical maps illustrate that *L1CAM* exhibits relatively higher expression in regions with larger CT (Pearson’s *R* = 0.35, *p* = 0.00048), whereas *UGT8* exhibits relatively lower expression in those regions (Pearson’s *R* = −0.37, *p* = 0.00017). **e** The number of the 1005 consensus CT-related PLS1 genes overlapped with those linked to CT in humans. *R* in **a**, **d** represent the Pearson correlation coefficient. *p* values in **a**, **d** are determined based on a two-sided test and not corrected for multiple comparisons. Each point in **a**, **d** stands for a cortical region (*n* = 97). Source data are provided as a Source Data file.

We next investigated the relationship between regional CT and anatomically patterned expression of cortical genes using PLS analysis, a multivariate statistical technique that decomposes relationship between two datasets into orthogonal sets of latent variables with maximum covariance and has been extensively used for neuroimaging and transcriptional data analysis^29,33,54^. With in-house generated structural MRI and transcriptomic datasets of cynomolgus macaques, we estimated CT of 97 cortical regions for 161 monkeys at age 2–8, and constructed a matrix (97 cortical regions × 23,613 genes) of transcriptional level values, and then subject to PLS regression for all age groups. The first component (PLS1) is defined as the spatial map that captures the greatest fraction of total expression variance across cortical areas, which explained 17.6–21.2% of the variance in our data (Supplementary Data 9), and significantly correlated with the CT values at each age group (Fig. 5a). In total, we found 2269 (positively or negatively weighted) PLS1 genes persistently associated with regional variations in CT from age 2 to age 8 (Fig. 5b). We next identified 1284 genes whose expression levels were significantly correlated with CT using Pearson correlation (*r* > 0.3, FDR corrected *p* < 0.05 at each age, Fig. 5c and Supplementary Data 10). As such, we determined consensus CT-related PLS1 genes as the overlapped ones between 2269 persistent PLS1 genes and 1284 CT-related genes, as led to 1005 genes including 532 positively (PLS1+) weighted genes (i.e., *PNCK*, *DOK4*, *TMEM130*, and *L1CAM*) and 473 negatively (PLS1−) weighted genes (i.e., *RCBTB1*, *ABCB7*, and *UGT8*) (Supplementary Data 11). Representative cortical maps of CT-correlated PLS1+ and PLS1− genes (e.g., *L1CAM* and *UGT8*) were illustrated in Fig. 5d. Of note, this set of 1005 genes consisted of 971 protein-coding genes, 18 long non-coding RNAs, 13 small RNAs and 3 pseudogenes. As a range of transcriptomic studies in human subjects have identified candidate genes associated with CT, we conducted a direct comparison with 1005 genes identified in the current study, and found that 94 of 1005 genes (OR = 1.43, *p* = 0.002, Fisher’s exact test) were significantly overlapped with those reported by Whitaker et al.^29^ (Fig. 5e and Supplementary Data 12).

### Cell-type and enrichment analysis of genes transcriptionally related to cortical thickness

**Fig. 6:**
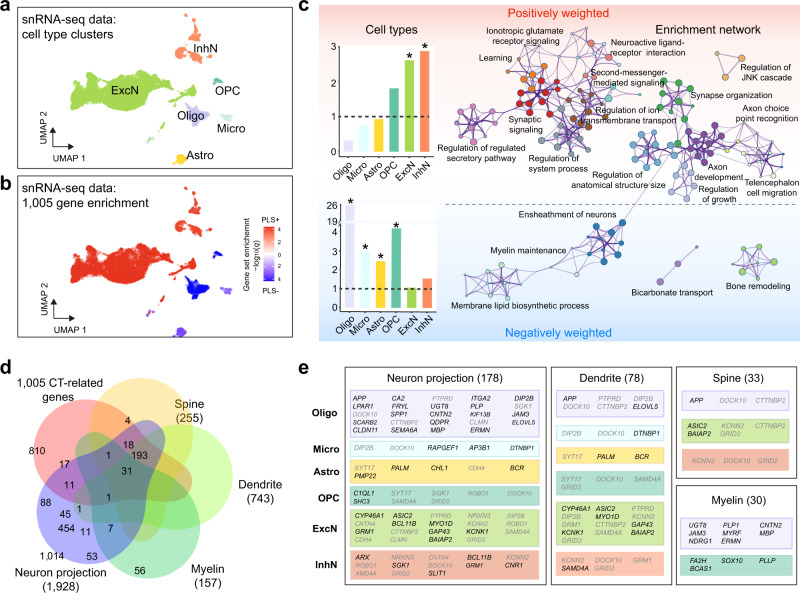
CT-related genes associated with specific cell types and involved in multiple biological processes. **a** UMAP visualization of six major cell type clusters identified in the cynomolgus macaque brain using single-nucleus RNA-sequencing (snRNA-seq) data. The classification of the single nuclei with each color represents a pre-defined cell type. Oligo oligodendrocyte, Micro microglia, Astro astrocyte, OPC oligodendrocyte precursor cell, ExcN excitatory neuron, InhN inhibitory neuron. **b** The enrichment of CT-correlated PLS1+ (red) and PLS1− (blue) genes within cells of each type is represented by color. **c** Enriched cell types and networks in CT-correlated PLS1+ and PLS1− genes, respectively. Left: The bar plots display the enrichment of positively and negatively weighted CT-related genes in the marker genes of six cell types. *p* values are determined based on two-sided Fisher’s exact test and provided in Supplementary Data 13. The asterisks denote odds ratio > 1 (y-axis) and FDR corrected *p* < 0.05. Right: Metascape enrichment network visualization shows the intra-clusters and inter-clusters of enriched functional terms. Each term is represented by a circle node, with the circle size proportional to the number of genes it contains. **d** The number of overlapping genes between 1005 CT-related PLS1 genes, neuron-projection related genes and three associated gene panels (spine, dendrite, myelin) according to Parker et al. **e** Typical genes related to specific cell types in each panel. The gene number profiled for each panel in 1005 CT-related genes is shown. Source data are provided as a Source Data file.

To identify cell types expressing genes correlated to cortical imaging phenotypes in cynomolgus macaque, we performed uniform manifold approximation and projection (UMAP) and clustering analyses on our V1 snRNA-seq data and focused on six interested cell clusters including oligodendrocytes, microglia, astrocytes, oligodendrocyte precursor cells (OPCs), excitatory and inhibitory neurons (Fig. 6a). For each cell type, marker genes were calculated by comparing the mean expression of each gene in this cell type against the mean of average expression in all other cell types based on a strict cutoff (FDR corrected *p* < 0.05, fold change ≥ 2). Subsequently, these cell type annotations were applied to enrichment analysis of 534 PLS1 + and 473 PLS1- genes, respectively (Fig. 6b). Contrasting one cell type with the others, CT-related PLS1 + genes were significantly enriched in excitatory (31, OR = 2.61, *p* = 7.81 × 10^−6^, FDR corrected for all comparisons below, Fisher’s exact test) and inhibitory (22, OR = 2.88, *p* = 2.95 × 10^−5^) neuron markers (Fig. 6c and Supplementary Data 13). Meanwhile, CT-related PLS1- genes were significantly expressed in oligodendrocytes (115, OR = 25.91, *p* = 8.41 × 10^−102^), microglia (13, OR = 2.92, *p* = 9.72 × 10^−4^), astrocytes (24, OR = 2.44, *p* = 1.76 × 10^−4^) and OPCs (21, OR = 4.15, *p* = 2.18 × 10^−7^). To further validate the results of cell type annotation, we replicated the present analyses using previously published single-cell/nucleus RNA-seq data acquired from the neocortex of cynomolgus macaques^6^ and the frontal region in rhesus macaques^55^ (Supplementary Fig. 11 and Supplementary Data 13). Sensitivity analyses also support this cell type enrichment (Supplementary Fig. 12). Additionally, similar enrichments were obtained by using cell-level enrichment methods, such as AUCell^56^ (Supplementary Fig. 13).

We aligned the gene ontology (GO) biological functions and KEGG pathways with consensus CT-related PLS1+ and PLS1− genes using Metascape^57^. After correcting for multiple enrichment comparisons (*p* < 0.05), the CT-related PLS1+ genes were significantly enriched for biological processes related to neuronal functions, such as “synaptic signaling”, “regulation of ion transmembrane transport”, “synapse organization”, and “neuroactive ligand-receptor interaction” whereas PLS1- genes were enriched for biological processes such as “myelin maintenance”, “ensheathment of neurons”, and “membrane lipid biosynthetic process” (Fig. 6c and Supplementary Data 14). Nevertheless, we used three prior CT-related gene panels of spine, dendrite and myelin^32^, and gene panel of neuron projection to validate these GO terms and benchmark the components of CT (Fig. 6d). It showed that 1005 CT-related PLS1 genes were highly overlapped with spine (33, OR = 3.42, *p* = 1.37 × 10^−8^), dendrite (78, OR = 2.78, *p* = 1.88 × 10^−13^), myelin (30, OR = 5.44, *p* = 4.02 × 10^−12^), and neuron projection (178, OR = 2.57, *p* = 1.42 × 10^−23^) associated genes (Fig. 6e, Supplementary Fig. 14 and Supplementary Data 15). Notably, the CT-related PLS1- genes were solely enriched in myelin-related genes (28, OR = 11.20, *p* = 9.08 × 10^−19^) (Supplementary Fig. 15). Moreover, most of the 1005 genes were retained and the same cell type enrichment and biological functions were observed when imaging-transcriptomics analyses were performed taking account of age and sex effects (Supplementary Figs. 16–18).

## Discussion

We applied large-scale bulk RNA-seq data on anatomically defined areas of the entire cynomolgus macaque brain to chart a complete transcriptional landscape in 3D MRI coordinate space, which opens new horizons for single-cell/nucleus and spatial transcriptomics in primates. Essentially, we observed similar regional expression profiles between macaque and human brain in terms of the number of expressed (80.50% vs 76–86.1%) and differentially regulated (59.98% vs 70.9–81.8%) genes, striking differences in transcriptional profiles between cortical and subcortical regions, and unique molecular signatures of the occipital lobe across the neocortex^10,48^. Moreover, we observed the full range of transcripts with their compositions varying across the whole brain, which thus enables the detection and the characterization of novel transcripts like long non-coding RNAs and alternative transcripts of protein-coding genes in future investigations. Using this transcriptomic atlas, one can examine topographic expression organization of specific gene sets in the macaque brain such as serotonin-signaling-associated genes, and accurately quantify regional enrichment of any single gene and plot it onto an MRI-derived brain map for visualization, which may serve as a reference catalog for developing exploratory study design and hypothesis generation. As an immediate example, identification of DEGs in distinct cell types within various brain regions of macaque monkeys, together with further elucidation of specific promoters/enhancers for expressing these genes, is a stepping stone to the development of molecular targets for cell-specific manipulation. This is rather attractive as viral-based transgenic and gene-editing approaches used in generating rodent models have been progressively extended to nonhuman primates^15,58–60^.

While incredible amounts of transcriptomics data at multiple spatial scales are now being aggregated from nonhuman primates and human brains, the transcriptional substrates that influence the anatomical/functional differentiations and specializations of the primate prefrontal cortex remain largely unknown^22,61^. Prior MRI studies leveraged the knowledge of twin-based heritability to deduce the macroscale genetic patterning of brain morphology including CT and surface area, both of which formed orthogonal axes to each other in cortical organization (dorsal–ventral axis versus anterior–posterior axis)^25,26^. Importantly, genetic contributions to CT and surface area has been reported prominently distinct^3,25^. Moreover, using neuroimaging data of human twins and macaque monkeys, the macroscale organization principle of genetic influences on CT was found comparable in primates^21^, indicating a phylogenetically conserved trait. Here we used alternative approaches of WGCNA analysis and clustering based on the neurotransmitter signaling associated genes to examine the topographic relationship of transcriptional variations between subdivisions of the frontal lobe. A mosaic transcriptomic pattern was observed in the frontal lobe, which was consistent with the broad subdivision networks in the prefrontal cortex described by Saleem et al.^62^ and topographically distinguished by a dorsolateral-ventromedial gradient. To a certain extent, this spatially varying pattern of expression profiles resembles the sensorimotor-to-transmodal gradient in the human cerebral cortex^12^. Interestingly, hub genes and enriched functional annotations for the dorsal module have been linked to axonal loss and circuit dysfunction of the motor cortex, thereby shedding light on genetic predisposition to neurodegenerative diseases^63,64^.

As a highly heritable trait, genetic influences on regional variations in the cortical thickness of the human brain have been extensively investigated under a variety of conditions. In our study, we found a constant relationship between regional morphological differences and gene expression. Among 1005 genes identified related to CT, some genes have been reported involved in structural components of myelin (*CNP*, *MOBP*, *PLLP*, *MOG*, *MAL*), synthesis of myelin constituents (*ASPA*, *UGT8*), regulation of myelin formation (*DOK4*, *KLK6*), oligodendrocyte differentiation (*ERBB3*) and transcription factors (*SOX10*) regulating other myelination-related genes^65–68^. Prior studies have found a significant amount of myelin reside as small bundles of fibers that run within the layers of the cortical gray matter based on ex vivo myelin-stained sections and MRI-estimated myelin volume fraction map in the superficial brain^69,70^, and this cortical myelination presumably speeds the conduction of signals to the input layer of areas that require fast responses. Moreover, the overlap of myelination-related genes and hub genes (i.e., *CNP*, *PLP1*, *UGT8*) of the module enriched in motor areas -- highly myelinated primary cortices, supports the myelin content map in primates observed with MRI contrasts^22,71^.

Rapid advances in spatial transcriptomic analysis now allow mapping of gene expression topography over the whole brain at single-cell/nucleus resolution. Identification of stably or uniquely expressed genes would help to define a cell type and further integrate transcriptome-based and connectome-based subtype classification as they may constitute a unique connectome pattern. Cell-type-specific contributions to spatial variation of CT with reference to cell type-specific signature genes have been assessed in human subjects^27^. Here we observed some of the consensus CT-related genes were associated with multiple aspects of neuronal function whereas others were enriched for oligodendrocytes and OPCs, which may explain an intricate relationship between CT and intracortical myelination^13,29,37^. It has been proposed that mature oligodendrocytes generate myelin sheaths that speed up nerve impulse conduction and provide metabolic support to axons, while OPCs generate myelinating oligodendrocytes throughout life, and likely serve yet-to-be-identified roles in circuit formation and function^72^. These results indicate that myelination-related genes may be evolutionarily conserved and mediate the biological processes linked to molecular and structural architectures in both macaque and human brains.

There are several practical limitations to this study when interpreting our results. Firstly, the present snRNA-seq data was solely sampled from V1 where the gene expression profiles are strikingly different from the rest of cortical regions. Therefore, we have utilized two recently published snRNA-seq data to cross-validate the cell type annotations for the frontal modules and consensus CT-related genes. Moreover, we performed sensitivity analyses for each individual cell type across the whole brain. Importantly, we identified V1 specific cell type markers such as *DNAH5*, *ABO* and *CNR1* (Supplementary Fig. 19) when performing subtype enrichment in excitatory and inhibitory neurons. Secondly, given the current lack of spatially fine-grained cortical expression in developing monkeys, we determined consensus CT-related genes by persistently tracking regional variation of CT regardless of their ages. It calls for future investigation of identifying candidate genes that may selectively drive CT variations at specific brain regions during certain developmental period. Nevertheless, we verified this set of CT-related 1005 genes by taking account of the effects of age and sex. Thirdly, although the current work focused mainly on one of the MRI-derived phenotypes, with the present transcriptome atlas, one is allowed to explore new possibilities such as the relationship between structural connectivity and gene expression at a fine spatial scale. Here, we presented an example which demonstrates the spatial correspondence between the tracer-derived structural connectivity matrix and regional gene co-expression (Supplementary Fig. 20). Importantly, the spatial mapping of the present transcriptome data can be readily converted to any other parcellation schemes for the primate brain^73^, especially when one is interested in integrating with other data modalities or probing cross-species comparison questions in one common stereotactic space^74^.

In short, this study presents a comprehensive transcriptomics resource for the macaque brain based on region-specific bulk RNA-seq in MRI coordinates, which enables an integrative analysis of single-cell/nucleus RNA-seq and multimodal brain imaging data. Given phylogenetic and central nervous system developmental proximity between nonhuman primates and humans, the present analysis strategy demonstrates a broadly applicable roadmap of probing interspecies evolutionary mechanisms from an imaging-transcriptomics perspective.

## Methods

### Animals

Nine adult cynomolgus monkeys (*Macaca fascicularis*; mean ± s.d., 13.6 ± 7.8 years, 8 males and 1 female) weighing 4.2–12.0 kg (8.6 ± 2.6 kg) were used for the study. All animal experimental procedures were approved by the Animal Care and Use Committee of CAS Center for Excellence in Brain Science and Intelligence Technology, Chinese Academy of Sciences, and conformed to National Institutes of Health guidelines for the humane care and use of laboratory animals.

### Brain tissue collection and RNA sequencing

A complete description of animal handling procedures can be found in our previous work^40,75–77^ and is briefly summarized here. Animals were euthanized with an overdose of ketamine (30–40 mg/kg) and isoflurane (3–5%), and perfused transcardially with ice-chilled sucrose-based artificial cerebrospinal fluid containing 234 mM sucrose, 2.5 mM KCl, 1.25 mM NaH_2_PO_4_, 10 mM MgSO_4_·7H_2_O, 0.5 mM CaCl_2_·2H_2_O, 26 mM NaHCO_3_, and 11 mM D-(+)-glucose^61,78^. Brains were immediately extracted and transferred into liquid nitrogen until frozen. Frozen brains were serially cryosectioned into coronal slices (2 mm thick) and placed on freezing, barcoded plates for storage at liquid nitrogen temperature. A small quantity of tissue samples (~100 mg/sample) were collected from anatomically distinct areas in each slice according to the brain atlas of cynomolgus macaques^40^ and then photographed by a digital camera, which allowed to identify the specific location (coordinates) of sampled areas with reference to MRI images of the monkey brain. Dissected tissue was used to extract total RNA using Trizol (Invitrogen) following the manufacturer’s directions. Note that all surgical instruments were treated with Diethyl pyrocarbonate for overnight to remove RNase and sterilized in advance. During the tissue collection, RNaseZap (Thermo Fisher Scientific) was carefully applied to the surface of surgical instruments for inactivation of exogenous RNases. Quality and quantity measurements of the extracted RNA were performed using NanoDrop (Thermo Fisher Scientific) and a Qubit Fluorometer (Thermo Fisher Scientific), respectively, and RNA integrity numbers (RIN) were determined using a Bioanalyzer RNA 6000 Nano Kit (Agilent, USA). Samples that passed the criteria of RIN ≥ 7 and RNA concentration ≥ 80 ng/μl were kept for subsequent bulk RNA-seq.

A paired-end sequencing library was designed on the Illumina Paired-end 150 sequencing platform. For each sample, 15G of data were generated by bulk RNA-seq. A total of 878 tissue samples were acquired across 111 regions defined by the brain template of cynomolgus macaques.

### Single-nucleus sample preparation

We isolated nuclei for dissected tissues sampled from the primary visual cortex by transferring to microcentrifuge tubes, snap frozen in a slurry of dry ice and stored at −80 °C until the time of use. Frozen tissues were initially treated with a homogenization buffer that consisted of 10 mM Tris pH 8.0, 250 mM sucrose, 25 mM KCl, 5 mM MgCl_2_, 0.1% Triton X-100 (Sigma-Aldrich), 0.5% RNasin Plus RNase inhibitor (Promega), 1× protease inhibitor (Promega), and 0.1 mM DTT (Sigma-Aldrich). Tissues were placed into a 1 ml dounce homogenizer (Wheaton) and homogenized using 11 strokes of dounce pestle to liberate nuclei. Homogenate was strained through a 30 mm cell strainer (Miltenyi Biotech) and centrifuged at 900 × *g* for 10 min at 4 °C to pellet nuclei. Nuclei were then resuspended in staining buffer containing 1× PBS (Sangon Biotech) supplemented with 0.8% nuclease free BSA (BioVision) and 0.5% RNasin Plus RNase inhibitor. Mouse anti-NeuN antibody Alexa Fluor 555 conjugated (Millipore, MAB377A5) was added to the nuclei at a dilution of 1:1000 and nuclei suspensions were incubated at 4 °C for 30 min at dark place. After incubation in the antibody, nuclei suspensions were centrifuged at 400 × *g* for 5 min and resuspended in clean staining buffer. Prior to fluorescence-activated cell sorting, 4′6-diamidino-2-phenylindole was applied to nuclei suspensions at a final concentration of 0.1 mg/ml and nuclei suspensions were filtered through a 35 mm nylon mesh to remove aggregates. Single-nucleus sorting was carried out on a BD MoFlo XDP Cell Sorter instrument to exclude debris and doublets. Single nucleus from neuron cells were collected by gating on Alexa Fluor 594 (NeuN) positive signal, nonneuronal nucleus were collected by gating on NeuN-negative signal. Strip tubes containing isolated single nuclei after cell sorting were then briefly centrifuged, removed the supernatant and diluted into 300 nuclei/µl using 1× PBS, 0.8% BSA, and 0.5% RNasin Plus. NeuN-positive nuclei and NeuN-negative nuclei were combined using a volume ratio of 9:1 and sent to sequencing center for 10x Chromium Single-Cell v3 loading following 15 cycles of cDNA amplification.

### Read alignment and quality control

High-quality reads (with averaged sequencing depth 52.8 million reads per sample) were mapped to the cynomolgus macaque genome Ensembl *M. fascicularis* (version: Macaca_fascicularis_5.0). FASTA and annotation files were downloaded from the Ensembl database (https://www.ensembl.org)^79^. This ensembl annotation comprises a total of 29,324 genes, including 21,584 protein-coding genes, 738 long non-coding RNAs, 6700 small RNAs (small non-coding RNAs and miscellaneous RNAs), and 302 pseudogenes.

Adapters of all sequenced reads were firstly trimmed by Trim Galore (version 0.6.0) with parameters: *-a AGATCGGAAGAGCACACGTCTGAACTCCAGTCAC -a2 AGATCGGAAGAGCGTCGTGTAGGGAAAGAGTGT --paired --stringency 3 --fastqc --phred33*. Then fastx_trimmer (FASTX Toolkit version 0.0.14) was used to cut the first 10 bp of reads to remove poor quality sequences. The clean reads were aligned to the gene assembly using STAR (version 2.7.3a)^80^ with command line “*—runMode genomeGenerate*” to build the sequence index. For example, the alignment of sample A is given below: *--runMode alignReads --runThreadN 16 –genomeDir crab-eating-macaque-mfas5 --readFilesCommand zcat --readFilesIn sampleA_R1.fastq.gz sampleA_R2.fastq.gz --outFileNamePrefix sampleA --outSAMattributes All --outSAMtype BAM SortedByCoordinate --limitBAMsortRAM 62000000000 --outSAMunmapped Within*. The entire mRNA mapping information were wrapped in the BAM format alignments.

After read alignment, quality control analysis was implemented to remove samples with poor quality. The number and percentage of uniquely mapped reads were calculated for each sample. In addition, sequencing metrics, such as %High-quality Aligned Reads, %mRNA Bases, %Intergenic Bases, Median 5′ to 3′ Bias, GC (guanine-cytosine) dropout rate, and AT (adenine-thymine) dropout rate, were computed by using PicardTools (version 2.21.2, http://broadinstitute.github.io/picard/, commands *CollectAlignmnetSummaryMetrics, CollectRnaSeqMetrics, CollectGcBiasMetrics*). To detect outlier samples, a quality *z* score was calculated for each metric. Samples with low quality (% unique mapping <50%, *Z* > 2 for %Intergenic Bases, GC Dropout rate, or AT Dropout rate and *Z* < −2 for %High-quality Aligned Reads, %mRNA Bases, or Median 5′ to 3′ Bias) in this matrix were identified as outliers, and any sample with greater than two outlier indexes was removed^20^. Thus, 52 of the initial 878 samples were removed after performing quality control procedures.

The number of reads uniquely mapped to each gene were counted using featureCounts^81^. And the gene expression level was quantified by RNA-Seq by Expectation Maximization (RSEM) to get transcripts per million^82^. Afterwards, *removeBatchEffect* function of limma package was used to remove batch effects^83^. Based on the normalized expression values, principal component analysis (*plotPCA* in DESeq2 package) and hierarchical clustering analysis (*hclust* function in stats package) were performed to visualize the relations among all RNA-seq samples. This analysis also identified additional 7 samples as outliers that were excluded. Hence, the remaining 819 samples spanning 110 brain regions (757 samples from 100 cortical areas and 62 samples from 10 subcortical areas, pooling across hemispheres) were used for evaluating the gene expression (Supplementary Data 1). Genes were considered robustly expressed if they are expressed in ≥10% samples. Thus, 23,605 and 23,613 genes (80.50% and 80.52% of 29,324 annotated genes) were obtained across whole-brain areas and in the cortical regions, respectively. The Pearson correlation coefficient between any pair of samples was calculated to construct an 819 × 819 expression matrix.

### Differential expression analysis

Using DESeq2 package^84^, pairwise differential expression gene (DEG) analysis was performed between any two brain regions which had at least three records of sequencing data passing quality control to improve the statistical power, including 102 discrete brain structures that comprised of 94 cortical regions and 8 subcortical areas for subsequent analysis. Notably, dense sampling over the entire brain allowed to uncover the subtle transcriptome-wide molecular-structural differences. The GC-content that was controlled by using conditional quantile normalization package^85^ was incorporated into the differential expression analysis. The reported *p* value was corrected for multiple testing using the Benjamini–Hochberg procedure to estimate the false discovery rate (FDR). Genes with fold change ≥2 and FDR-corrected *p* < 0.05 was identified as DEGs. Moreover, genes retained across different combinations of minimum count and minimum number of samples were examined (Supplementary Fig. 16).

Pairwise differential exon usage (DEU) analysis was conducted between any pair of brain regions using Subread_to_DEXSeq package. First, routine dexseq_prepare_annotation2.py was run to produce a featureCounts-readable GTF file. Second, count reads were quantified using the GTF file of cynomolgus macaque and then submitted to the DEXSeq package for DEU analyses^85^. Statistical significance level was adjusted for multiple testing using Benjamini-Hochberg to estimate the FDR and only significant DEUs with FDR-corrected *p* < 0.05 was used for further analysis.

### Visualization of neurotransmitters expression patterns

Gene panels related to 8 neurotransmitters (serotonin, acetylcholine, dopamine, epinephrine, gamma-aminobutyric acid (GABA), glutamate, glycine and norepinephrine,) were created in the light of previous human results^85^. Then the expression value for a given gene in each anatomical region was averaged and the median expression value was subtracted, followed by an exponential transformation (2^x^) between different genes. We retained 3 brain regions (24a, 36c, TF) with at least two samples to better visualize the whole-brain expression pattern. The MATLAB function *bar3* was used for brain visualization.

### Weighted gene co-expression network analysis

Following the standard procedures of weighted gene co-expression network analysis (WGCNA)^49,50^, we constructed co-expression network based on normalized cortical gene expression data. Briefly, we filtered for protein-coding genes, lncRNAs and microRNAs and hence excluded the bottom 5% lowly expressed genes from further analysis. It gave rise to 19,971 genes from cortical regions that were used to estimate pairwise correlations using the biweight midcorrelation^86^. Then, co-expression modules consisting of positively correlated genes with high topological overlap were identified based on the signed weighted correlation matrix (*blockwiseModules* function in WGCNA package, softpower = 12)^50^. Modules were defined as branches of a hierarchical cluster tree using the dynamic tree cut method^87^ and the minimal module size was set to 50. Module eigengene (ME), defined as the first principal component of the expression matrix, was used to represent the expression pattern for each module. Pairs of similar modules (ME correlation *r* > 0.8) were merged and 20 modules were identified.

These identified modules were characterized with several ways. First, modules were annotated by gene ontology (GO) and Kyoto Encyclopedia of Genes and Genomes (KEGG) pathway via g:Profiler^88^. Second, modules were annotated by over-represented analysis for cell-type high expression genes and neurotransmitter-related genes. Neurotransmitter genes were derived from a previous human study^8^. Third, the Pearson correlation between each gene and each ME was calculated to construct the module network. Hence hub genes with high gene-module eigengene correlation (*k*_ME_) for each module were visualized.

### MRI datasets and data processing

The structural MRI dataset of 162 cynomolgus macaques (3.5 ± 1.8 years, 72 females and 90 males) were collected on 3.0T MRI scanners at the Institute of Neuroscience (*n* = 29) and Kunming Institute of Zoology (*n* = 133), Chinese Academy of Sciences, as described in our recent work^40^. High-resolution T1-weighted anatomical images of macaque brain were acquired with key parameters as follows: TR = 2300 ms; TE = 3 ms; inversion time = 1000 ms; flip angle = 9°; acquisition voxel size = 0.5 × 0.5 × 0.5 mm^3^. Five to 7 whole-brain anatomical volumes were recorded for each subject. After preprocessing of monkey MRI data^40,77^, the thickness of the macaque cerebral cortex sheet was estimated for each subject using the diffeomorphic registration-based CT approach, which is a reliable volume-based technique for estimating voxel- and regional-wise thickness information (http://stnava.github.io/ANTs/)^89^ and yields similar results as that using surface-based algorithms^90^. To perform imaging-transcriptomics analysis in all age groups, we averaged the CT of each region for animals at the same age and obtained 7 imaging variables ranging between 2 and 8 years (only one subject at age 9 was excluded hence). As such, data from a total of 161 monkeys were used in this study.

### Imaging-transcriptomics analysis

Here MRI-derived CT in each region was considered as response variables while several thousands of genes in each region was used as predictor variables. The first PLS component (PLS1) was the linear combination of gene expression values that was most strongly correlated with regional CT, and the statistical significance of the PLS1 explained variance was evaluated by permuting the response variables 10,000 times. Bootstrapping was used to estimate the error of each gene’s PLS1 weight (resampling with replacement of the 97 cortical regions), and the ratio of the weight of each gene to its bootstrap standard error was used to calculate the Z-scores and rank the genes according to their contributions to PLS1. The gene sets with *Z* > 3 (PLS1+, positively weighted) or *Z* < −3 (PLS1−, negatively weighted) were considered CT-related. This procedure was repeated for different ages from 2 to 8, and genes that exhibited persistent associations with CT across all age groups were assembled as consensus PLS1 genes. In addition, we identified the gene list out of all cortical genes showing significant correlations with CT (|Pearson correlation coefficient| > 0.3, FDR-corrected *p* < 0.05 at each age, CT-correlated genes). To explore the contribution of CT-related genes in the PLS analysis, we obtained the overlapped genes from consensus PLS1 genes and CT-correlated gene list, which was named consensus CT-related PLS1 genes including positively (PLS1+) and negatively (PLS−) weighted genes. PLS1+ gene expression weights were positively correlated with variations in CT, indicating these genes were overexpressed in regions where CT was increased in monkeys. By contrast, PLS1− genes were overexpressed in regions where CT was decreased in monkeys.

To examine the effect of sex on our results, we replicated the above analyses using male-only imaging and transcriptome data. Furthermore, additional analyses were conducted to test the robustness of our results. Briefly, we fitted a linear model considering the effect of age for each region and regarded the sets of intercept and beta coefficients as inputs to the PLS. The regional intercept represents regional differences in CT and the beta coefficient for age captures the regional variation in the effects of age on CT. We also modeled the CT at each region as a function of age with male-only imaging data and put sets of intercept and beta coefficients as inputs to the PLS against the male-only transcriptomic data.

To better understand the biological significance of the positive and negative gene sets, Metascape database^57^ was used to conduct GO and KEGG pathway enrichment analyses. Both PLS1+ (*Z* > 3) or PLS1− (*Z* < −3) gene sets were input into the Metascape website (https://metascape.org/gp/index.html#/main/step1) and the obtained enrichment GO term and KEGG pathways (FDR-corrected *p* < 0.05) were visualized via enrichment network to show intra-cluster and inter-cluster similarities. To determine the biological processes including spine, dendrite, myelin^32^ and neuron projection in which consensus CT-related PLS1 gene sets are most involved, four gene panels were utilized: (1) spine panel contains genes related to spines structure and function; (2) dendrite panel contains genes related to structure and function of the entire dendritic arbor; (3) myelin panel captures genes associated with myelin structure and function; (4) neuron-projection panel includes genes related to structure and function of neuron projection (Supplementary Data 12). Specifically, human genes for each selected GO term were then downloaded from AmiGO2 website.

### Single-nucleus transcriptome data analysis

17,509 single nuclei acquired from the monkey cortex were processed using the default 10X Genomics CellRanger pipeline (Version 6.1.1). The reference genome was constructed based on Ensembl *M. fascicularis* using function *cellranger mkref*. And reads were mapped and processed by the function *cellranger count* to generate count matrix. The mean reads per nuclei were 47,133 and the median number of genes of nuclei was 3642. Nuclei with detected genes less than 500 and more than 7500 were removed for further analysis. The preprocessed gene expression data of 14,952 nuclei were then analyzed by the Seurat (version 4.1.0) package^91^ and the following steps were performed in order: data normalization and transformation, highly variable gene selection, principal component analysis (PCA) and clustering. The count matrix was first normalized and transformed using function *SCTransform*. The top 3000 highly variable genes were then obtained by *FindVariableGenes* with the default variance stabilizing process. We further embedded ensuing nuclei in the PCA dimensions followed by Uniform Manifold Approximation and Projection (UMAP) visualization^92^. Top 30 principal components were used for nuclei clustering and the resolution of *FindClusters* was set to 0.8.

Cortical cell classes were determined based on previously known cell type marker expression^17,93^. UMAP visualization and unsupervised clustering revealed six major canonical cortical cell classes including excitatory neurons, inhibitory neurons, microglia, astrocytes, oligodendrocytes, and oligodendrocyte precursors^94^. Marker genes were identified by comparing the mean expression of each gene in one cell type against mean of average expression in all other cell types by using function FindAllMarkers with the parameter *method* = *MAST* in Seurat package. To assess cell-type-specific enrichment of co-expression modules and identified consensus CT-related PLS1 gene sets, we considered genes expressed at least twofold higher in one cell type than all other cell types (FDR-corrected *p* < 0.05)^6^. Furthermore, highly expressed genes of distinct cell types from prior single-cell/nucleus macaque cortical data were used here for external validation^6,55^. The first dataset consists 11 cell types identified in the neocortex of *M. fascicularis*, including excitatory neuron, 6 subtypes of inhibitory neurons (*SST*+, *PVALB*+, *NYP*+, *LAMP5*+, *RLEN*+, *VIP*+), microglia, astrocyte, oligodendrocyte, OPC^6^. The second dataset consists 29 subtypes identified in dorsolateral prefrontal cortex (dlPFC) of rhesus macaque, including 10 subtypes of excitatory neurons, 9 subtypes of inhibitory neurons, 4 subtypes of glia cells and 6 non-neural subtypes^55^. The highly expressed genes of each cell type were identified by the same method used above. Furthermore, the R package AUCell was used to perform a cell-level enrichment of consensus CT-related PLS1 genes^56^.

### Gene ontology and enrichment analysis

GO and KEGG pathway enrichment analyses were conducted using g:Profiler and Metascape. The significant GO term and KEGG pathways with FDR-corrected *p* < 0.05 were reported. The gene set enrichment analysis was performed using a two-sided Fisher’s exact test with 95% confidence interval (R function *fisher.test*). Only significant enrichment with an odds ratio (OR) > 1 and FDR-corrected *p* < 0.05 were used.

### Reporting summary

Further information on research design is available in the Nature Portfolio Reporting Summary linked to this article.

## Supplementary information

Supplementary Information

Description of Additional Supplementary Files

Supplementary Data 1-15

Reporting Summary

## Data Availability

The RNA-seq data generated in this study have been deposited in the Sequence Read Archive (SRA) under accession code PRJNA905082. The public datasets used in this study can be accessed as described below: The single-cell RNA-seq data of macaque neocortex is available at https://db.cngb.org/nhpca/. The snRNA-seq data of the macaque dlPFC is available at http://resources.sestanlab.org/PFC/. The cynomolgus macaque genome Ensembl Macaca_fascicularis_5.0 is available at https://www.ensembl.org. D99 template of macaque brain is available at https://afni.nimh.nih.gov/pub/dist/atlases/macaque/. Subcortical atlas of macaque used for visualization can be downloaded from https://afni.nimh.nih.gov/pub/dist/doc/htmldoc/nonhuman/macaque_tempatl/atlas_sarm.html. The Cynomolgus macaque template (*Cyno162*) is available at 10.1093/cercor/bhaa229. Allen Human Brain Atlas data is available at https://human.brain-map.org/. Predicted comprehensive human mRNA expression data is available at http://www.meduniwien.ac.at/neuroimaging/mRNA.html. Results and statistics related to main figures are provided in Supplementary Data 1–15. Source data are provided with this paper.

## Source data

Source Data

## Code availability

Custom code for data analysis is available on a GitHub repository. The version of the code used in this study was archived in the Zenodo repository under the accession code 10.5281/zenodo.7641873.
