# Supplementary material for: Brain-wide and cell-specific transcriptomic insights into MRI-derived cortical morphology in macaque monkeys
Source: Nat Commun. 2023 Mar 17;14:1499. doi: 10.1038/s41467-023-37246-w (PMC10023667; doi:10.1038/s41467-023-37246-w)
Supplement: Supplementary file 1 — Supplementary Information [file 41467_2023_37246_MOESM1_ESM.pdf]

Supplementary Information for "Brain-wide and cell-specific transcriptomic insights into MRI-derived cortical morphology in macaque monkeys"

by Bo. et al.

Supplementary Figures

Supplementary Fig. 1 Region-level comparison of human and macaque bulk tissue RNA-seq data.
Supplementary Fig. 2 Cortical expression of serotonin-signaling-associated genes in human subjects.
Supplementary Fig. 3 Topography of expression distributions for major neurotransmitter systems across the entire brain.
Supplementary Fig. 4 Pairwise DEGs of 102 regions detected with edgeR and limma and comparison to DESeq2 methods.
Supplementary Fig. 5 Sample-level and region-level principal component analyses for characterizing gene expression features in different lobes/regions.
Supplementary Fig. 6 Correlation matrix of 20 WGCNA modules and 4 merged modules in frontal lobe.
Supplementary Fig. 7 Anatomical patterning of original 20 modules demonstrated on cortical surface.
Supplementary Fig. 8 Heatmaps showing similar distinct dorsolateral-ventromedial pairwised Spearman correlation variation in frontal lobe based on expression profiles of neurotransmitter and their receptors.
Supplementary Fig. 9 Illustration of gene-gene connections for module M1, M2, and M4.
Supplementary Fig. 10 Enrichment of major cell types of WGCNA modules (a-c) and 4 modules within the frontal lobe (d-f).
Supplementary Fig. 11 Cell type enrichment for 1,005 CT-related genes using snRNA-seq data from cynomolgus macaque and rhesus macaque.
Supplementary Fig. 12 Sensitivity analyses based on cell type marker genes of our snRNA-seq and two external datasets.
Supplementary Fig. 13 Cell cluster annotations and cell type enrichment of 1,005 CT-related genes annotated with three independent snRNA-seq datasets using AUCell.
Supplementary Fig. 14 Visualization of the positive and negative weighted genes in 1,005 CT-related genes (weights > 0.1).
Supplementary Fig. 15 Overlapping of CT-related PLS1 genes and gene panels of spine, dendrite, myelin, and neuron projection.
Supplementary Fig. 16 Genes retained across different combinations of minimum count and minimum number of samples.
Supplementary Fig. 17 Linear modelling the effect of sex and age.
Supplementary Fig. 18 Linear modelling of age and evaluation the PLS results in male-only data.
Supplementary Fig. 19 Cell subtype enrichment for 1,005 CT-related genes based on snRNA-seq data obtained from V1 and examples of cell type-specific genes.
Supplementary Fig. 20 Correspondence between tracer-, tractography-derived structural connectivity matrices and regional gene co-expression.

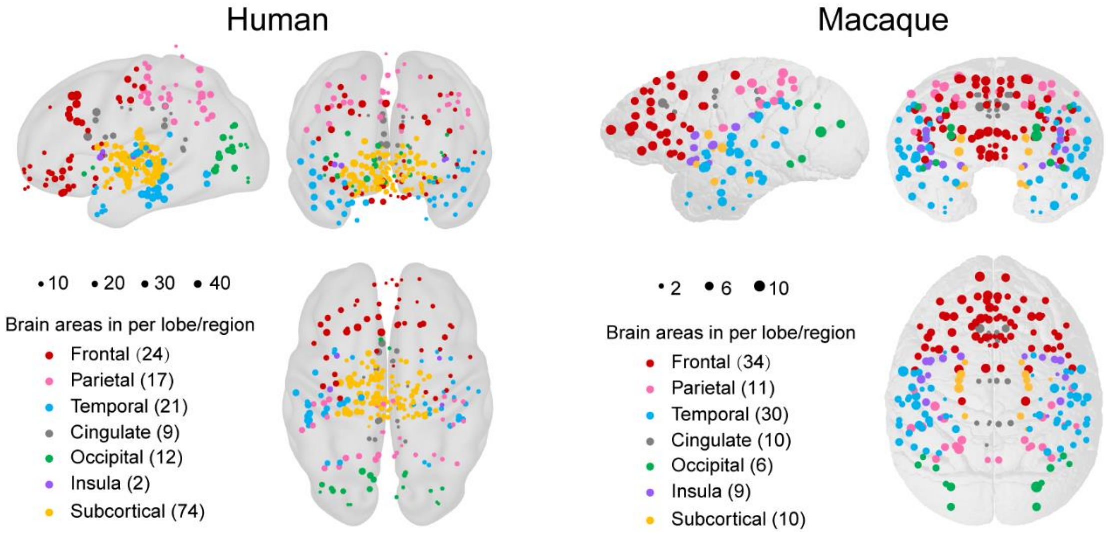

Supplementary Fig. 1 Region-level comparison of human and macaque bulk tissue RNA-seq data. Macaque transcriptomic data covered more subdivisions in frontal and temporal lobes compared to that in Allen Human Brain Atlas [1, 2, 3], pooling across hemispheres after strict quality control. Samples in cerebellum and brain stem were not included for visualization in human.

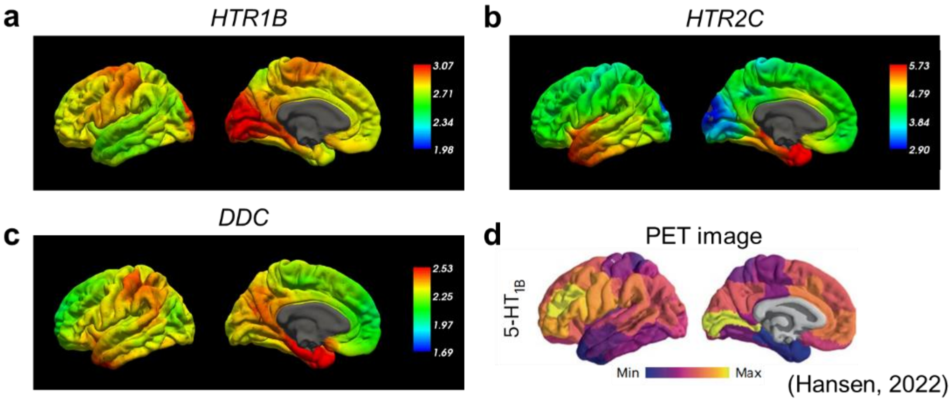

Supplementary Fig. 2 Cortical expression of serotonin-signaling-associated genes in human subjects. (**a-c**) Cortical map of mRNA expressions of serotonin receptor (*HTR1B* and *HTR2C*) and *L-Dopa-Decarboxylase* (*DDC*) predicted from human transcriptional data (download from the website: http://www.meduniwien.ac.at/neuroimaging/mRNA.html). **d** PET image of *HTR1B* as shown in a study that collected more than 1,200 healthy participants [4].

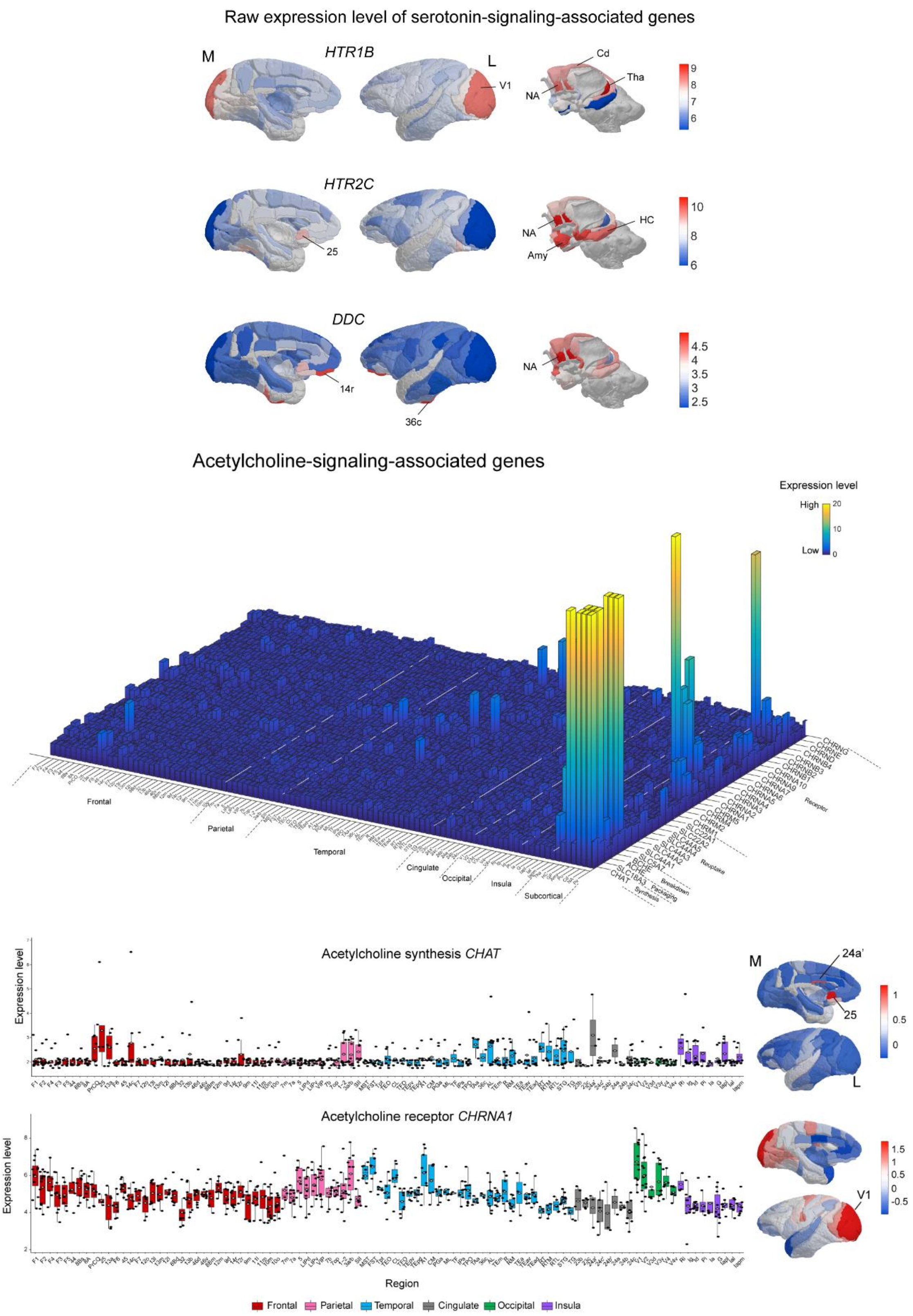
Raw expression level of serotonin-signaling-associated genes
HTR1B
M
L
V1
Cd
Tha
NA
HTR2C
25
NA
HC
Amy
DDC
14r
36c
NA
Acetylcholine-signaling-associated genes
Expression level
High
Low
Frontal
Parietal
Temporal
Cingulate
Occipital
Insula
Subcortical
Receptor
Reuptake
Breakdown
Packaging
Synthesis
Acetylcholine synthesis CHAT
Expression level
24a'
25
Acetylcholine receptor CHRNA1
Expression level
V1
Region
Frontal
Parietal
Temporal
Cingulate
Occipital
Insula

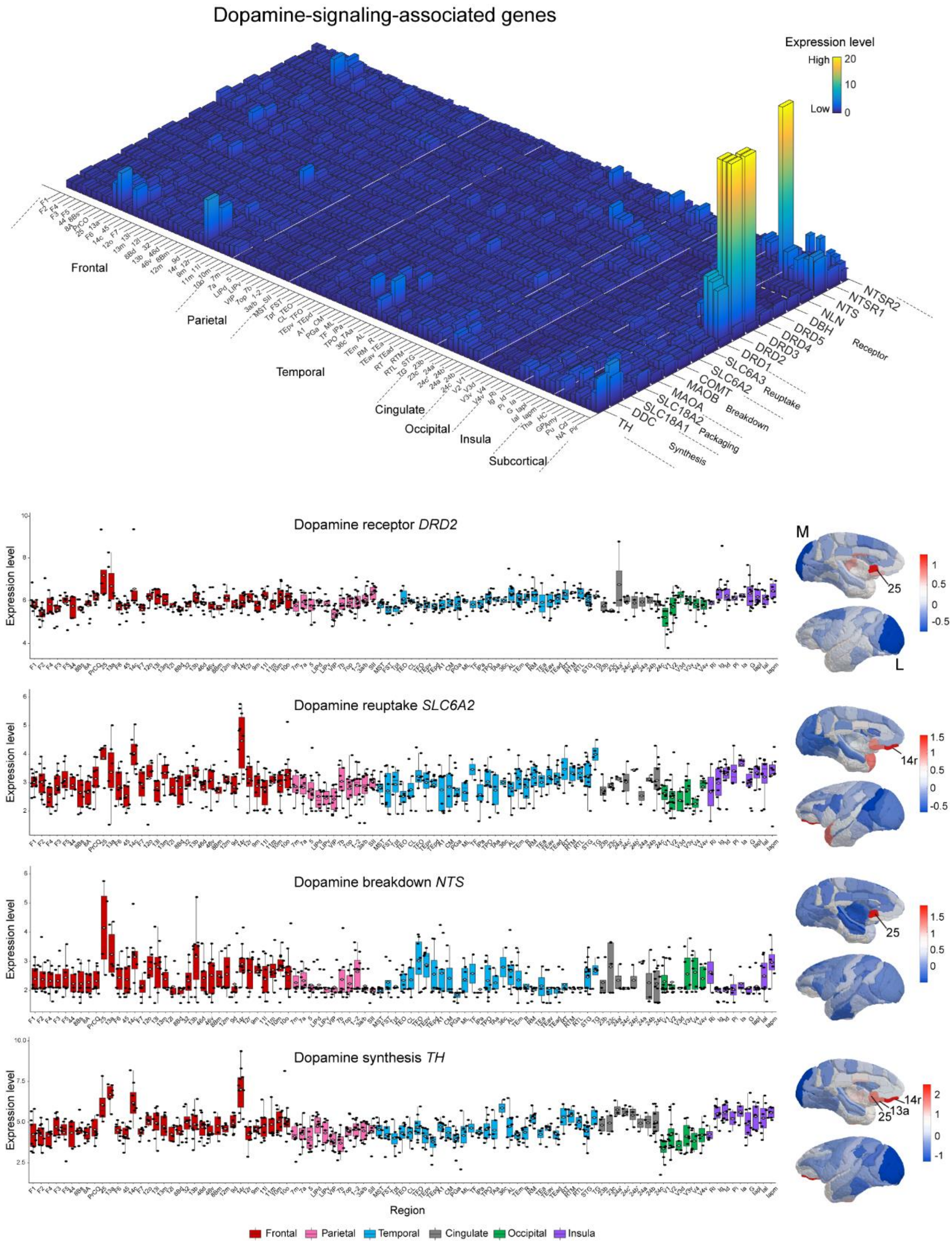
Dopamine-signaling-associated genes
Expression level
High
Low
20
10
0
Frontal
Parietal
Temporal
Cingulate
Occipital
Insula
Subcortical
TH
DDC
SLC18A1
SLC18A2
MAOA
MAOB
COMT
SLC6A2
SLC6A3
DRD1
DRD2
DRD3
DRD4
DRD5
DBH
NLN
NTS
NTSR1
NTSR2
Synthesis
Packaging
Breakdown
Reuptake
Receptor
Dopamine receptor DRD2
Dopamine reuptake SLC6A2
Dopamine breakdown NTS
Dopamine synthesis TH
Expression level
Region
M
L
25
14r
13a
Frontal
Parietal
Temporal
Cingulate
Occipital
Insula

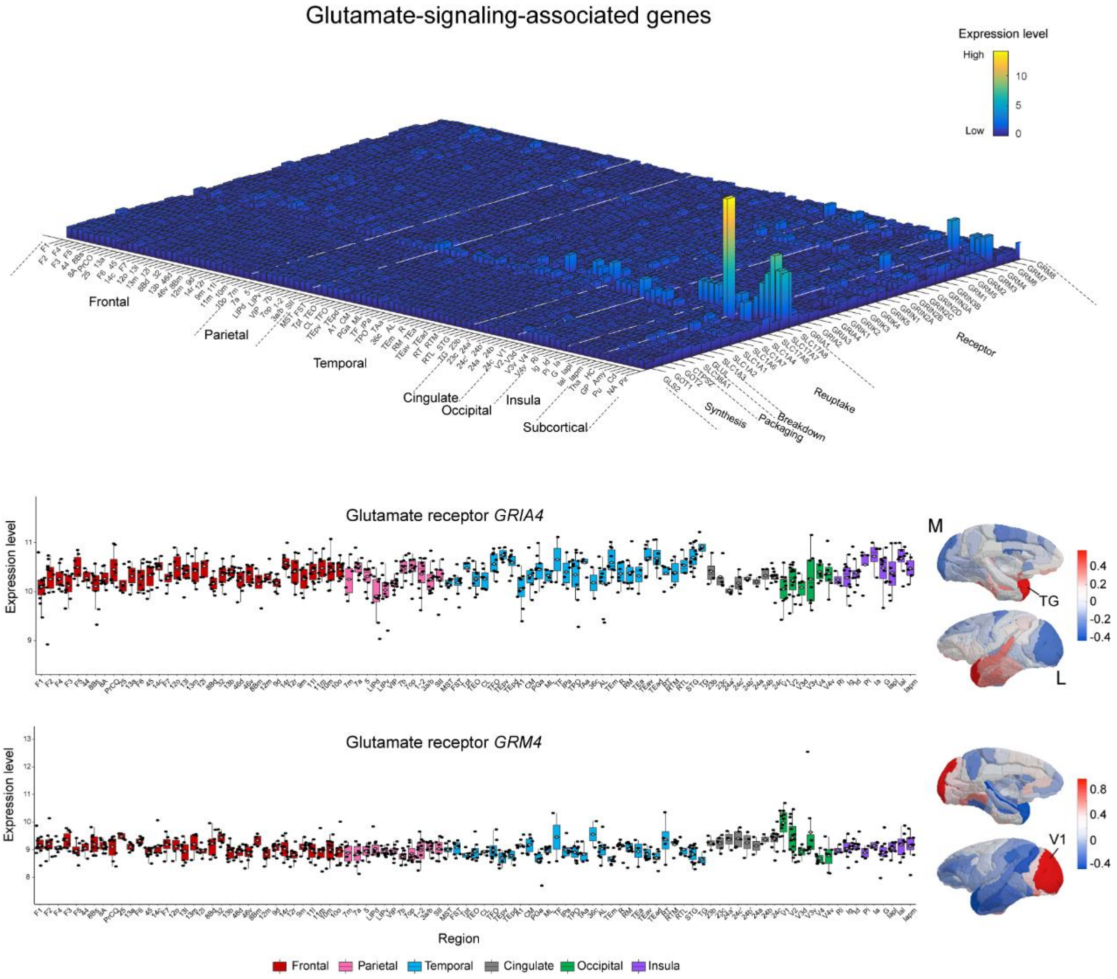
Glutamate-signaling-associated genes
Expression level
High
Low
Frontal
Parietal
Temporal
Cingulate
Occipital
Insula
Subcortical
Synthesis
Packaging
Breakdown
Reuptake
Receptor
Glutamate receptor GRIA4
Expression level
M
TG
L
Glutamate receptor GRM4
V1
Region
Frontal
Parietal
Temporal
Cingulate
Occipital
Insula

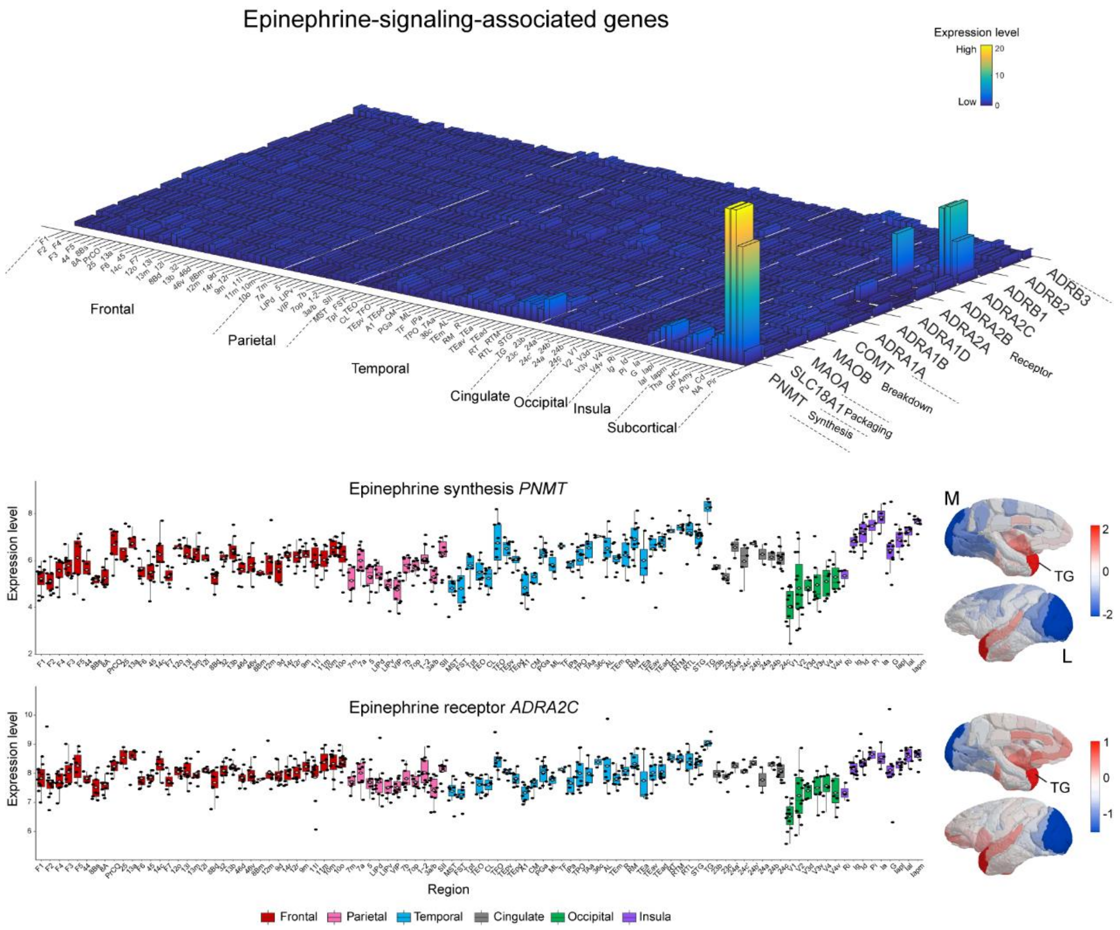
Epinephrine-signaling-associated genes
Expression level
High
20
10
Low
0
Frontal
Parietal
Temporal
Cingulate
Occipital
Insula
Subcortical
PNMT
SLC18A1
MAOA
MAOB
COMT
ADRA1A
ADRA1B
ADRA1D
ADRA2A
ADRA2B
ADRA2C
ADRB1
ADRB2
ADRB3
Synthesis
Packaging
Breakdown
Receptor
Epinephrine synthesis PNMT
Expression level
M
L
TG
2
0
-2
Epinephrine receptor ADRA2C
Expression level
Region
TG
1
0
-1
Frontal
Parietal
Temporal
Cingulate
Occipital
Insula

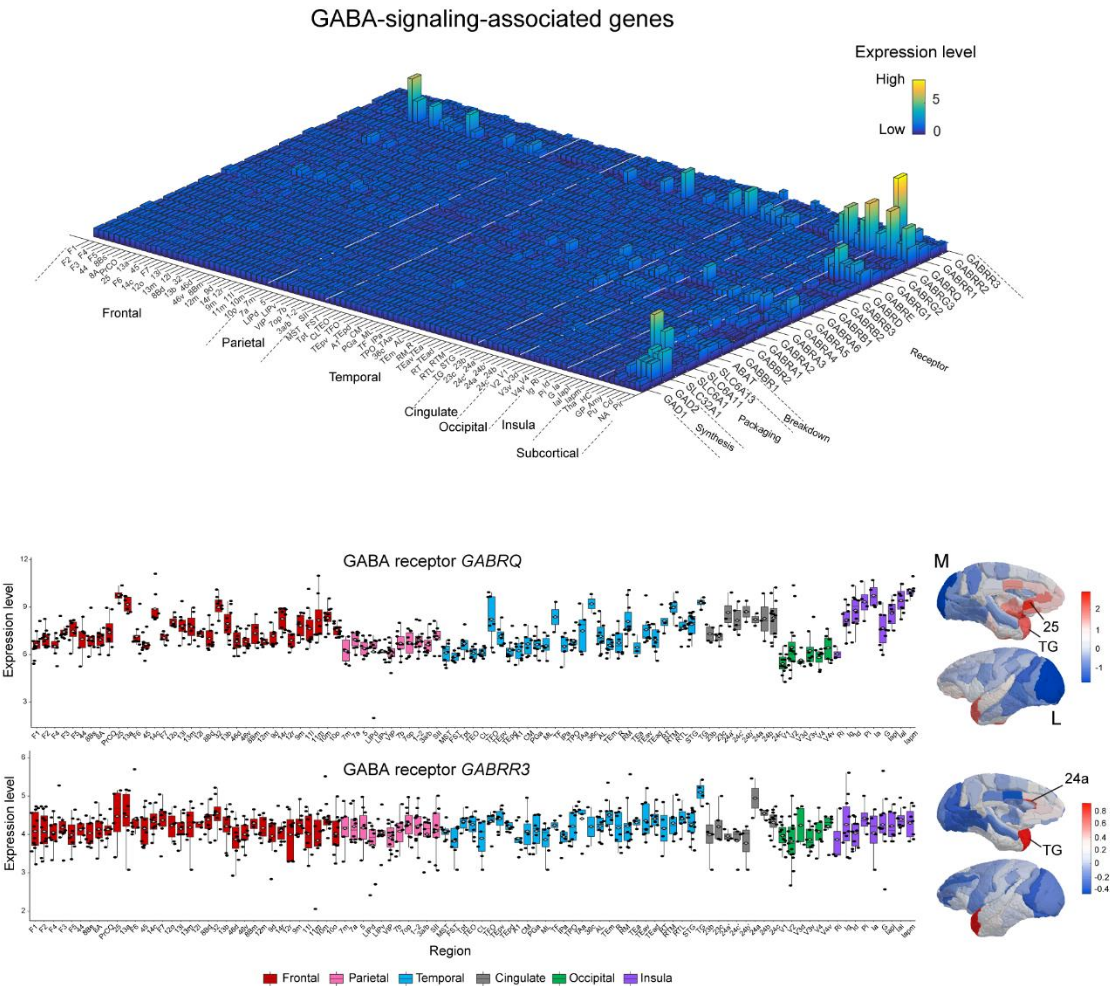

GABA-signaling-associated genes
Expression level
High
Low
5
0
Frontal
Parietal
Temporal
Cingulate
Occipital
Insula
Subcortical
Synthesis
Packaging
Breakdown
Receptor
GABA receptor GABRQ
GABA receptor GABRR3
Expression level
Region
M
L
25
TG
24a
Frontal
Parietal
Temporal
Cingulate
Occipital
Insula

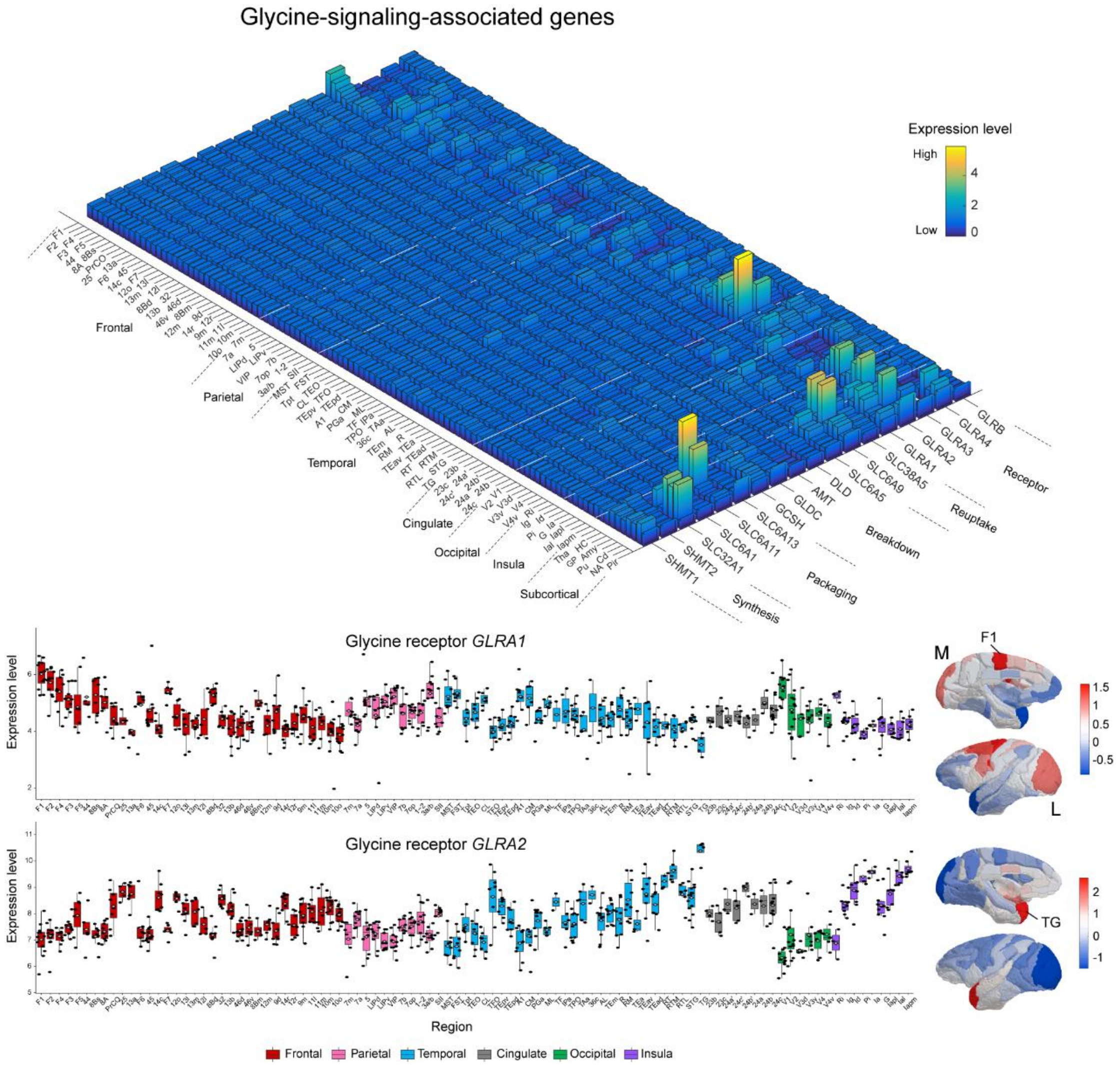

Glycine-signaling-associated genes
Expression level
High
Low
4
2
0
Frontal
Parietal
Temporal
Cingulate
Occipital
Insula
Subcortical
GLRB
GLRA4
GLRA3
GLRA2
GLRA1
SLC38A5
SLC6A9
SLC6A5
DLD
AMT
GLDC
GCSH
SLC6A13
SLC6A11
SLC6A1
SLC32A1
SHMT2
SHMT1
Receptor
Reuptake
Breakdown
Packaging
Synthesis
Glycine receptor GLRA1
Glycine receptor GLRA2
Expression level
Region
M
L
F1
TG
1.5
1
0.5
0
-0.5
2
1
0
-1
Frontal
Parietal
Temporal
Cingulate
Occipital
Insula

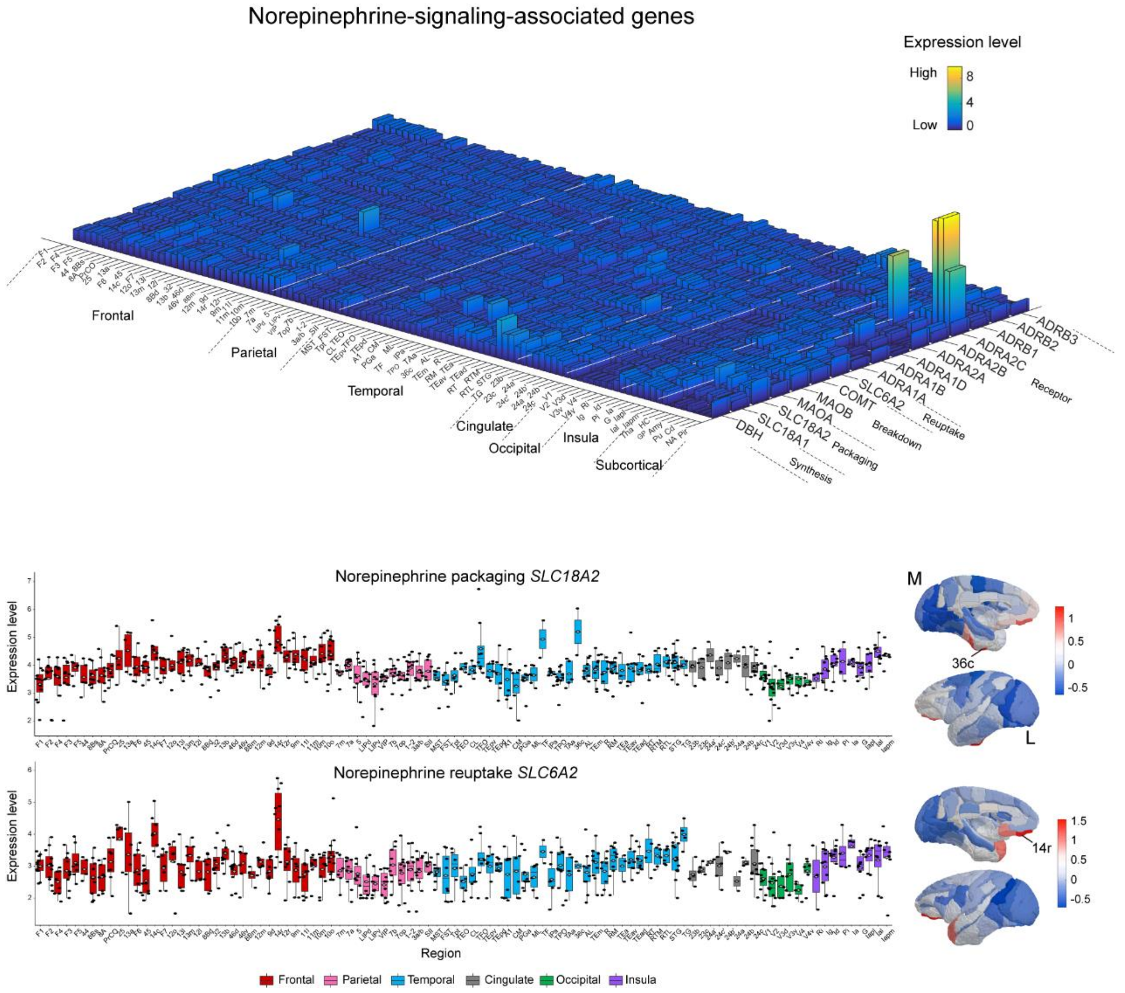

Supplementary Fig. 3 Topography of expression distributions for major neurotransmitter systems across the entire brain. Cortical and subcortical visualization of raw expression level of three serotonin-signaling-associated genes, and the expression topography of genes involved in synthesis, packaging, breakdown, reuptake and signaling of other major neurotransmitters (acetylcholine, dopamine, glutamate, epinephrine, GABA, glycine, norepinephrine). Normalized expression values were transformed to the same scale by subtracting the median value, followed by an exponential transformation. Highly expressed genes are marked at corresponding brain structures and the spatial expression pattern of these genes are shown on cortical surface and the normalized expression level across 97 cortical regions is plotted in the below. In each box plot, the center line indicates the median, the edges of the box indicate the 25th and 75th percentile (interquartile range, IQR) and the whiskers indicate last point within a 1.5x IQR (sample size varies among brain regions, ranging from 2 to 15). All sampled areas are color-coded by major structure; bars below from left to right are frontal, parietal, temporal, cingulate, occipital and insula. M, medial view; L, lateral view. See Supplementary Data 1 for structure abbreviations along x-axes. Tha, thalamus; Cd, caudate; NA, nucleus accumbens; Amy, amygdala; HC, hippocampus. Source data are provided as a Source Data file.

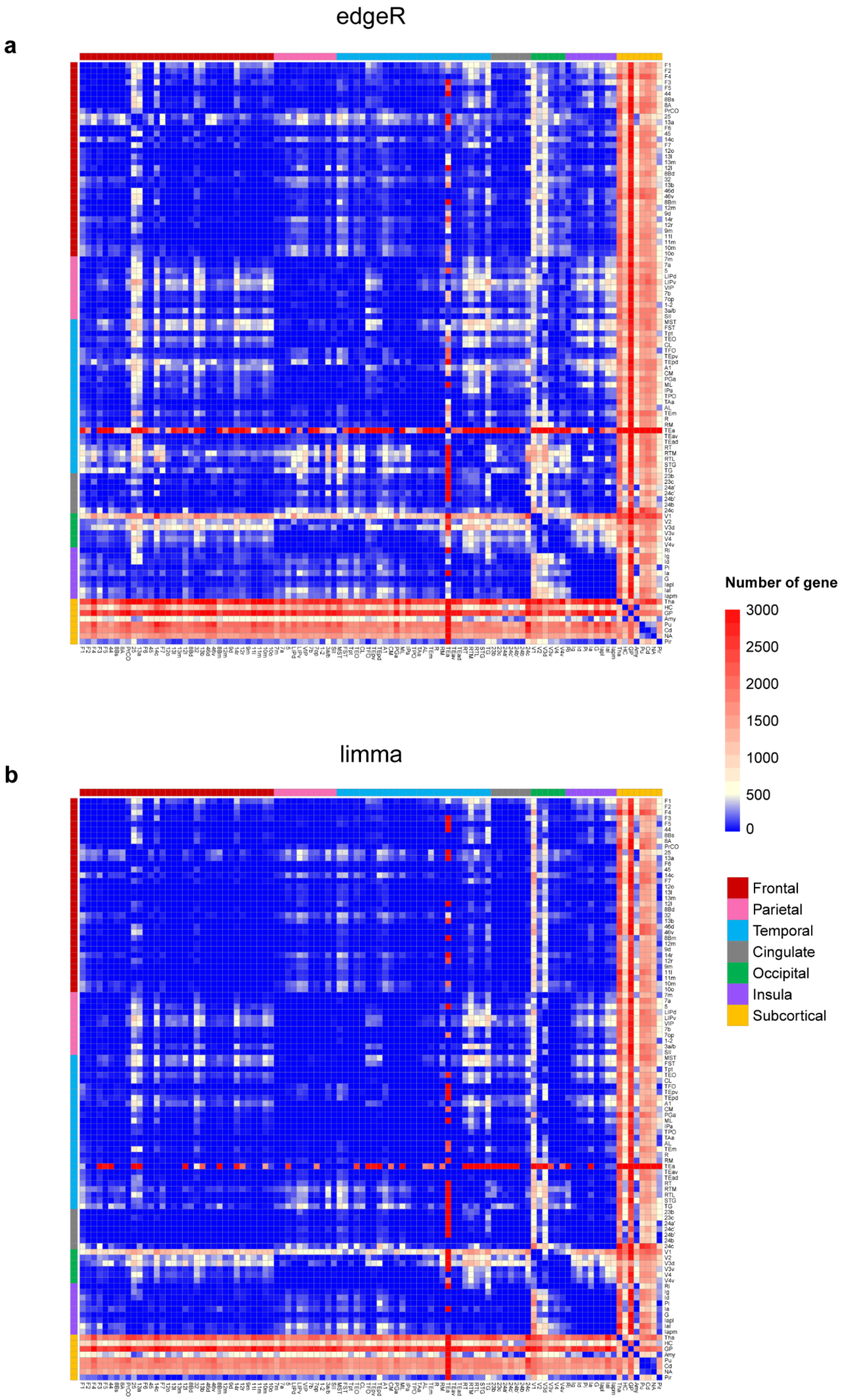
edgeR
a
limma
b
Number of gene
3000
2500
2000
1500
1000
500
0
Frontal
Parietal
Temporal
Cingulate
Occipital
Insula
Subcortical

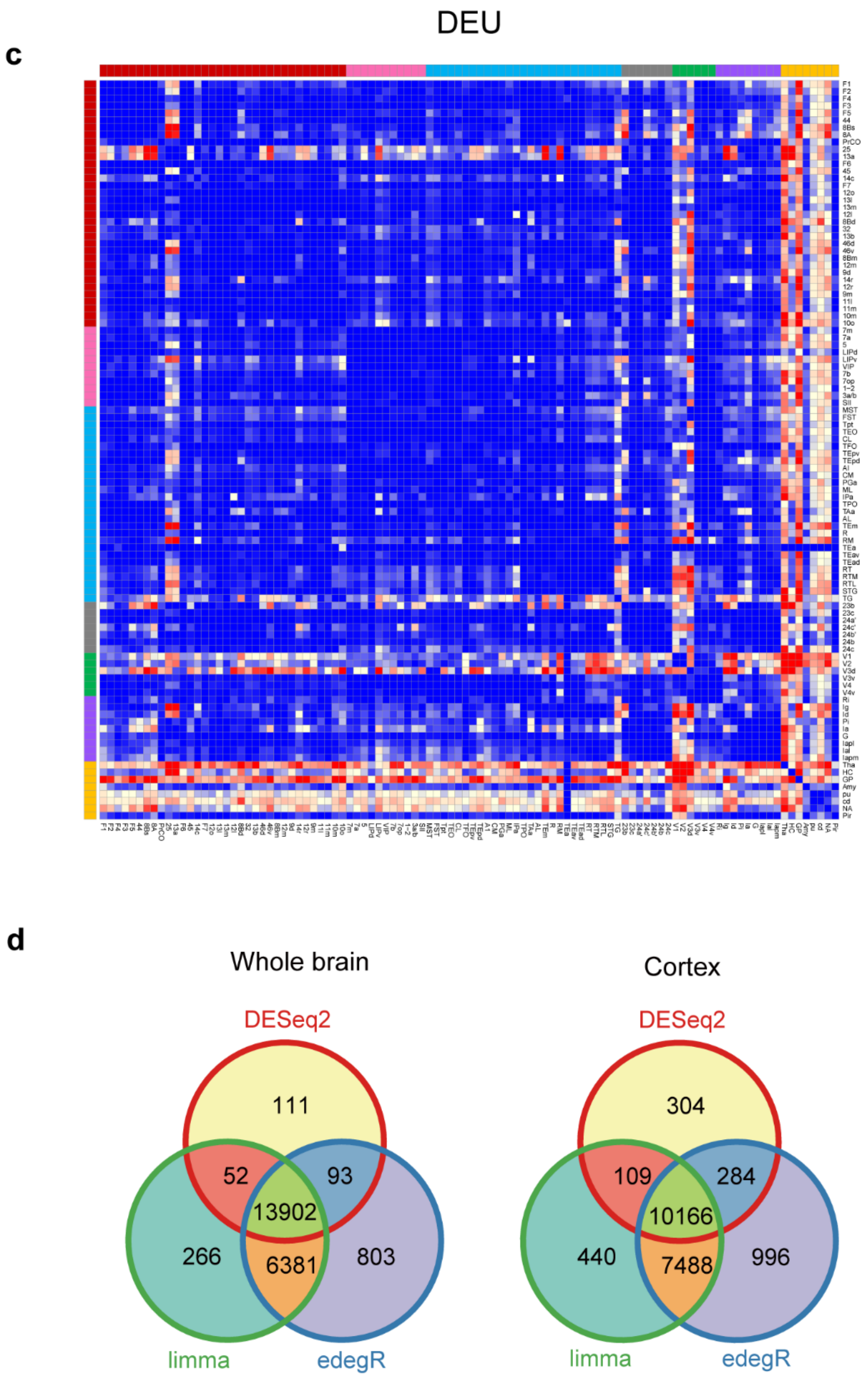

Supplementary Fig. 4 Pairwise DEGs of 102 regions detected with edgeR and limma and comparison to DESeq2 methods. Pairwise DEGs matrices detected by edgeR **(a)** and limma **(b)** methods and DEUs **(c)** matrix were shown. The matrix entry represents the number of genes up-regulated (bottom left) and down-regulated (top right) with a fold change > 2 in expression level. Similar to Fig. 3a, subcortical and occipital regions exhibit marked heterogeneous patterns (red) in contrast to the rest cortical regions (blue). **d** Brain-wide and cortex-wide overlapping of DEGs detected by DESeq2, edgeR and limma. See Supplementary Data 1 for a complete list of abbreviations for all sampled regions. Source data are provided as a Source Data file.

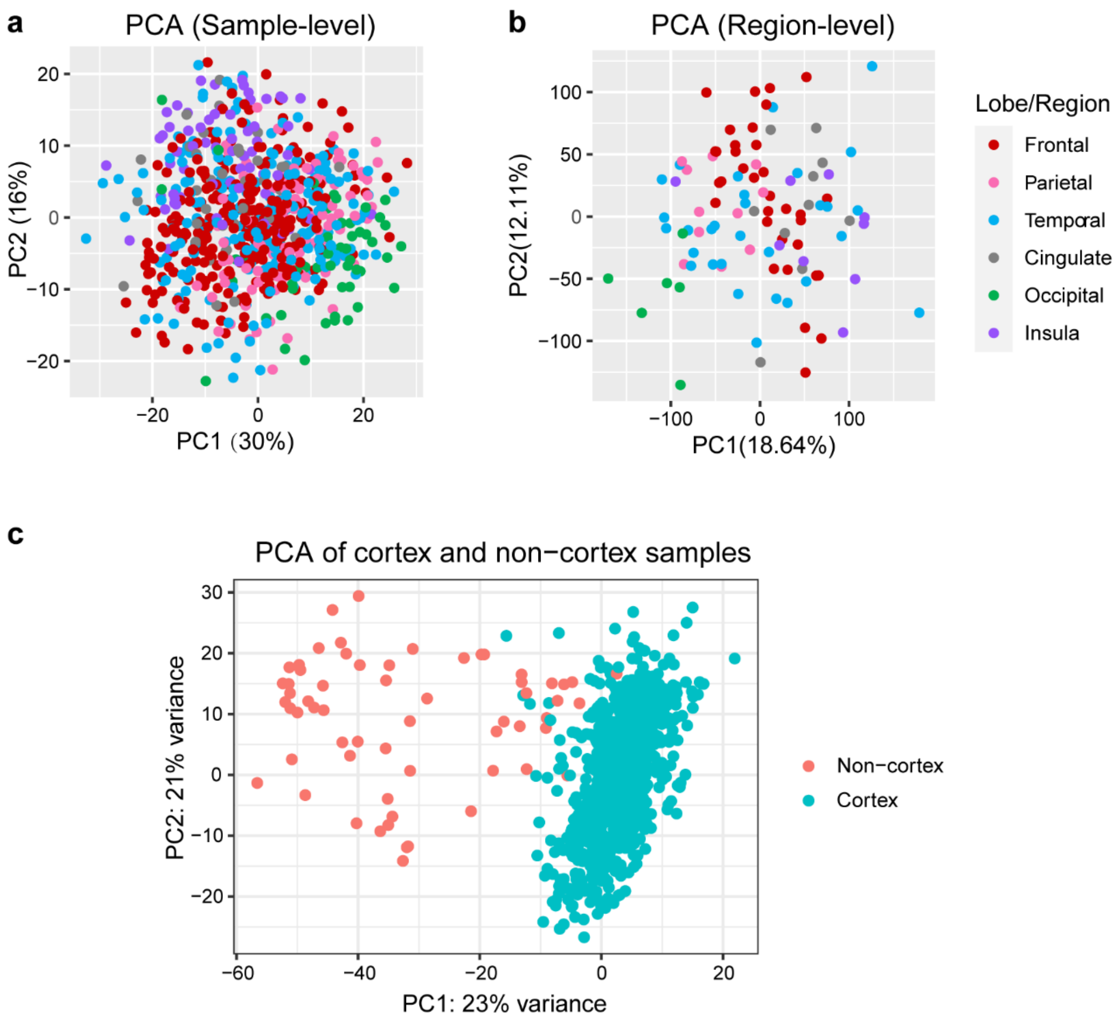

Supplementary Fig. 5 Sample-level and region-level principal component analyses for characterizing gene expression features in different lobes/regions. In contrast to the rest of cortical areas, the occipital lobe of macaque brain had the most distinctive transcriptional profile, reflected both in sample-level **(a)** and region-level **(b)** analyses. **c** PCA result also showed transcriptional differences between cortex and non-cortex samples. Source data are provided as a Source Data file.

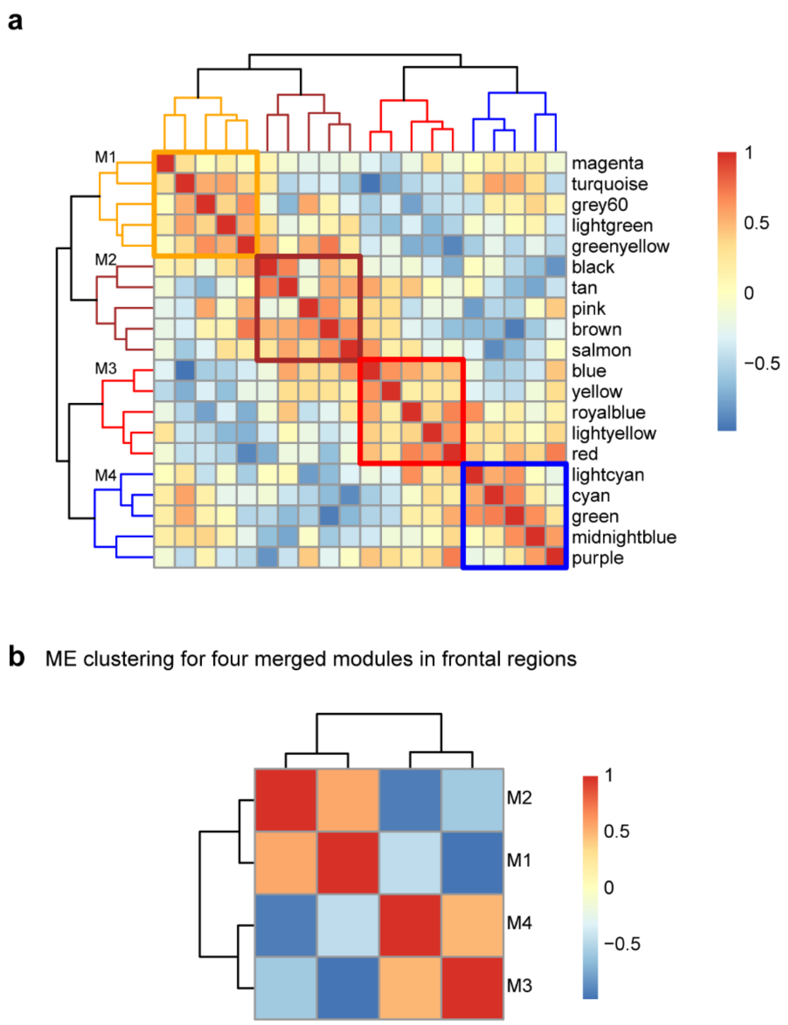

Supplementary Fig. 6 Correlation matrix of 20 WGCNA modules and 4 merged modules in frontal lobe. a Correlation heatmap of 20 WGCNA modules based on module eigengenes (ME) revealed four clusters: M1-M4. **b** Correlation heatmap of M1-M4 based on module eigengenes of frontal regions.

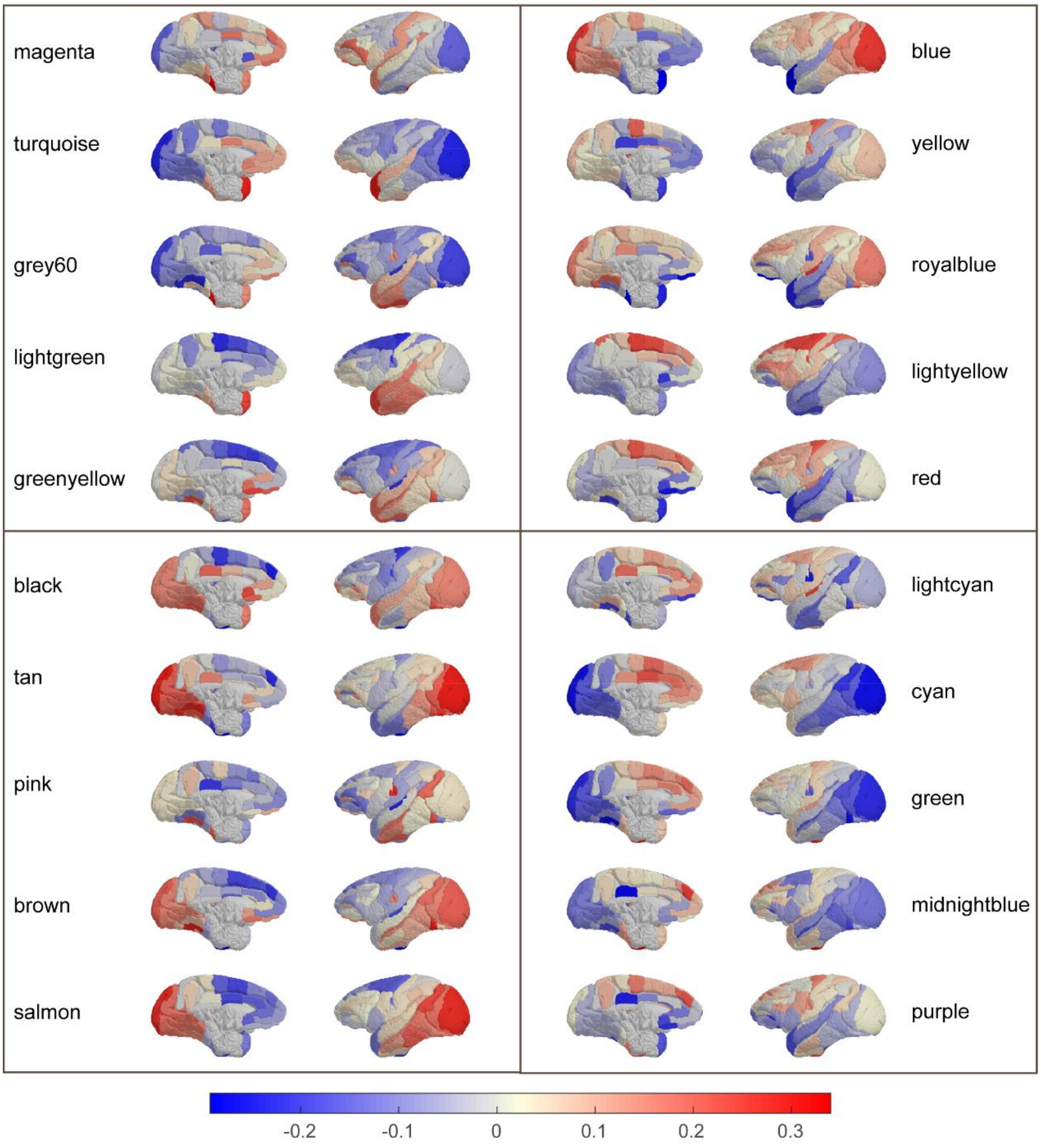

Supplementary Fig. 7 Anatomical patterning of original 20 modules demonstrated on cortical surface. Module eigengenes (ME) of 20 WGCNA modules were plotted on cortical surface.

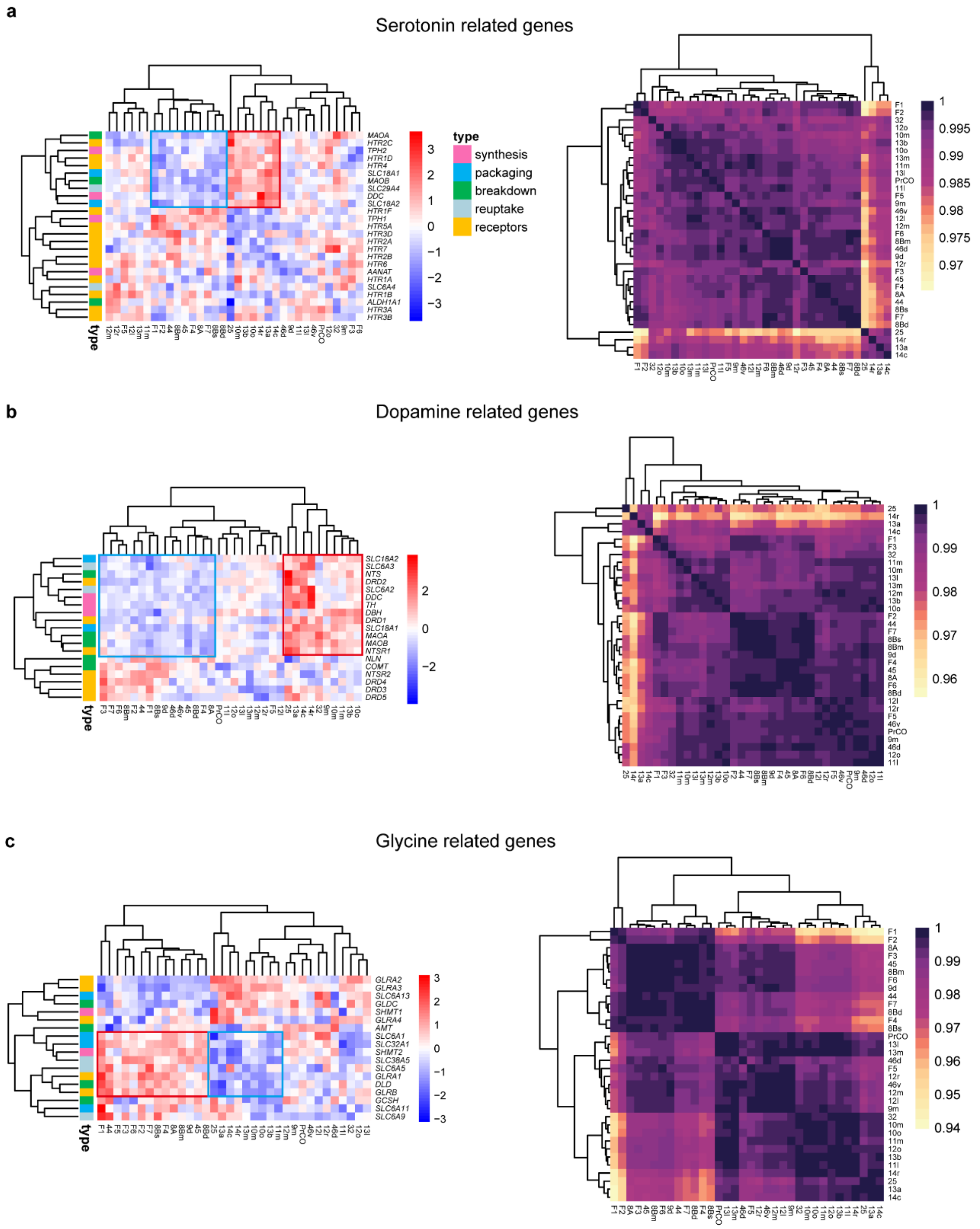

Supplementary Fig. 8 Heatmaps showing similar distinct dorsolateral-ventromedial pairwised Spearman correlation variation in frontal lobe based on expression profiles of neurotransmitter and their receptors. Serotonin (**a**), dopamine (**b**) glycine (**c**). Clustering in frontal lobe of other five neurotransmitters were provided in Supplementary Data 6.

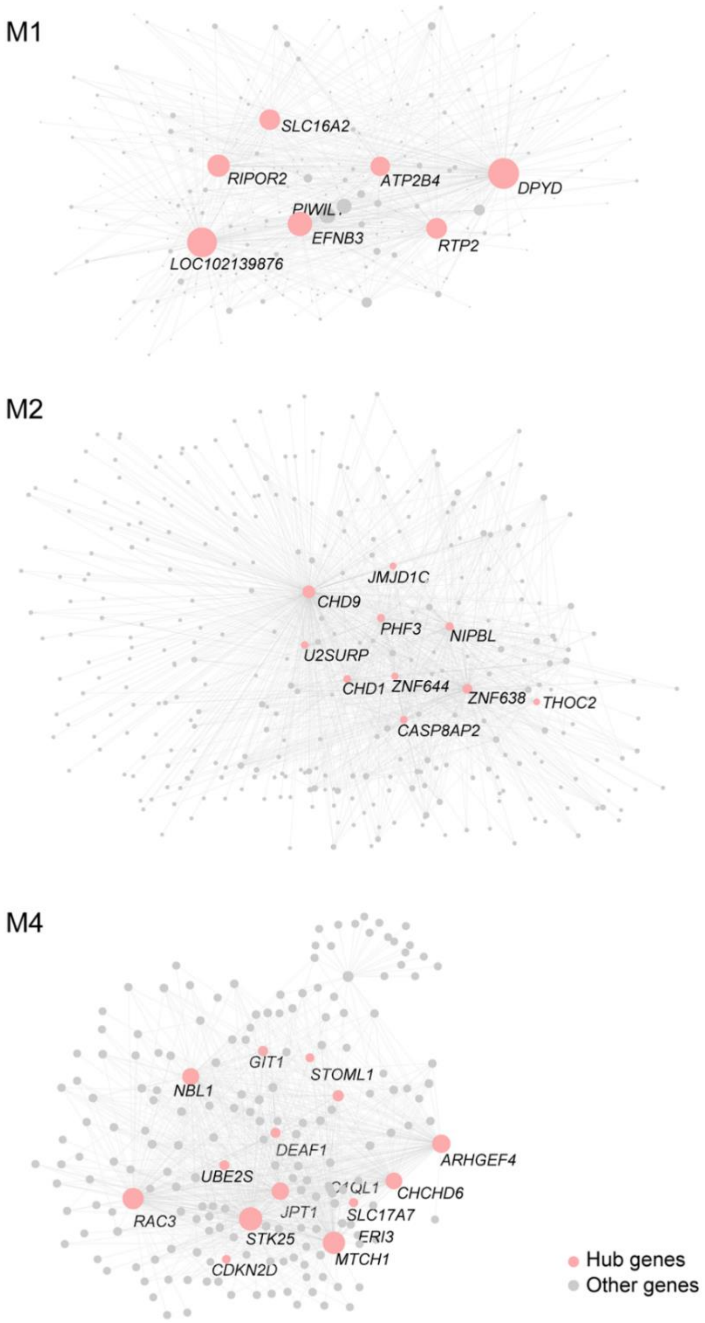

Supplementary Fig. 9 Illustration of gene-gene connections for module M1, M2, and M4. Hub genes are colored in pink. For better visualization, genes with different thresholds of intra-module expression similarity in each module (0.23 in M1, 0.13 in M2, 0.1 in M4) were artificially chosen to keep similar gene numbers with M3.

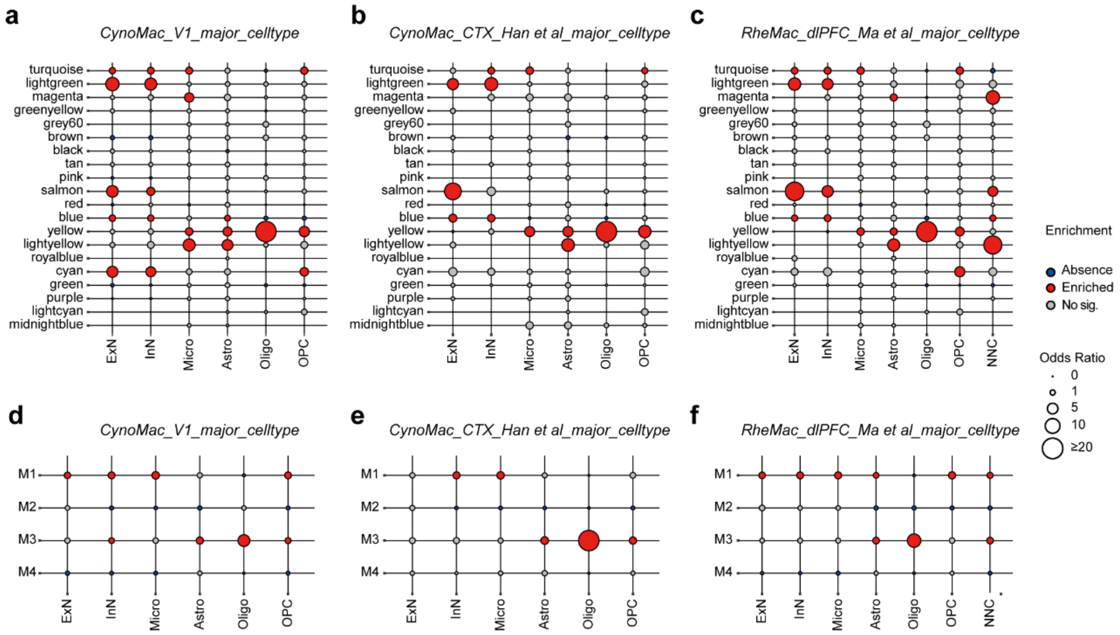

Supplementary Fig. 10 Enrichment of major cell types of WGCNA modules (a-c) and 4 modules within the frontal lobe (d-f). External validations were conducted with two independent snRNA-seq macaque datasets [5, 6].

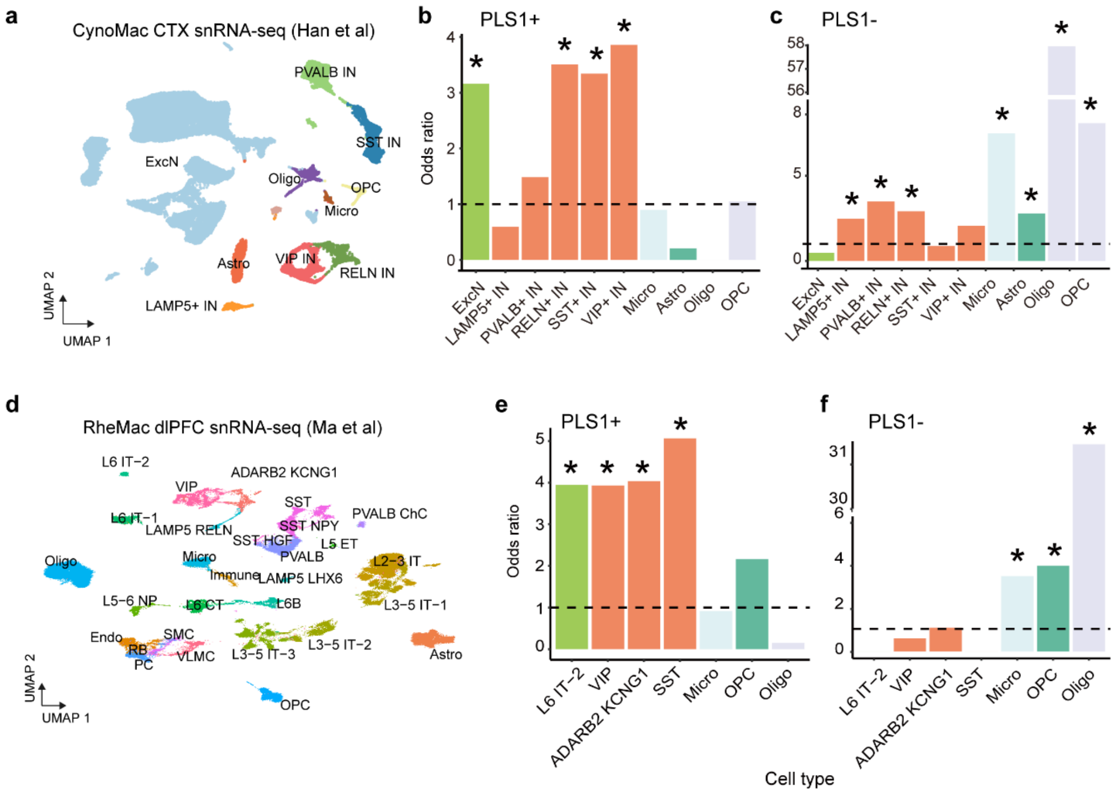

Supplementary Fig. 11 Cell type enrichment for 1,005 CT-related genes using snRNA-seq data from cynomolgus macaque and rhesus macaque. a UMAP visualization of different cell type clusters based on the marker genes from snRNA-seq data in cynomolgus macaque [5]. The classification of single cells with each color represents a pre-defined cell type. CTX, cortex. **b-c** Enriched cell types in CT-correlated PLS1+ and PLS1- genes respectively. **d** UMAP visualization of different cell type clusters based on the marker genes from snRNA-seq data in rhesus macaque (sampled from dlPFC) [6]. **e-f** Enriched cell types in CT-correlated PLS1+ and PLS1- genes respectively. *p*-values in **b**, **c**, **e**, and **f** are determined based on two-sided Fisher's exact test and the asterisks denote odds ratio > 1 (y-axis) and FDR corrected p < 0.05. Source data are provided as a Source Data file.

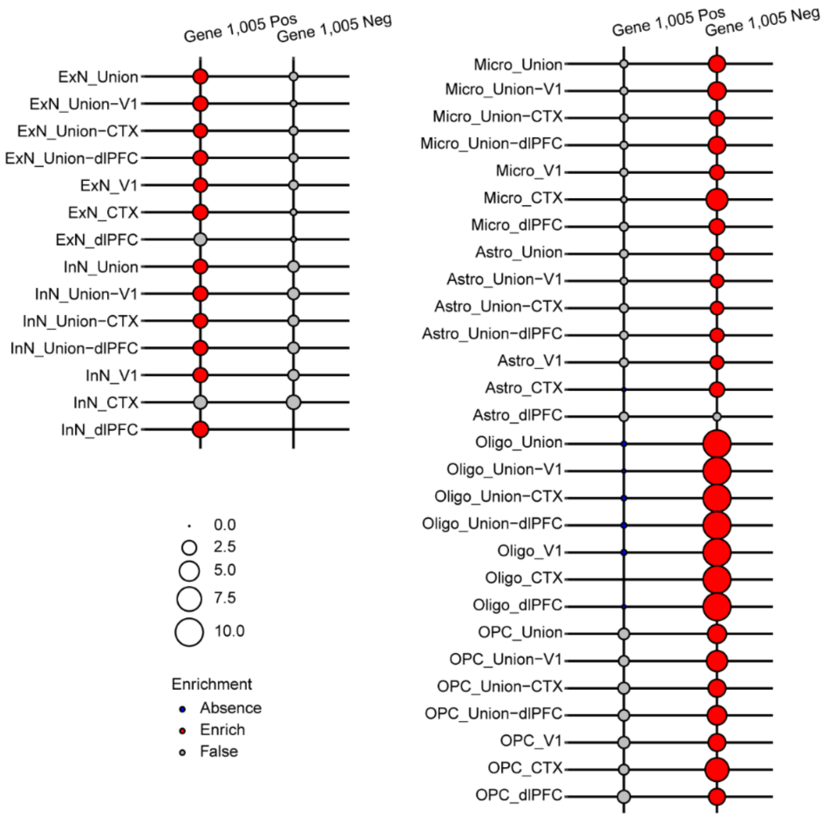

Supplementary Fig. 12 Sensitivity analyses based on cell type marker genes of our snRNA-seq and two external datasets. Union represents the shared marker genes of three snRNA-seq datasets and gene clusters named with union and the brain region represents the marker genes in each dataset, Union-V1, Union-CTX [5] and Union-dlPFC [6]. Moreover, gene clusters with the dataset name represent the specific marker genes only from that snRNA-seq data.

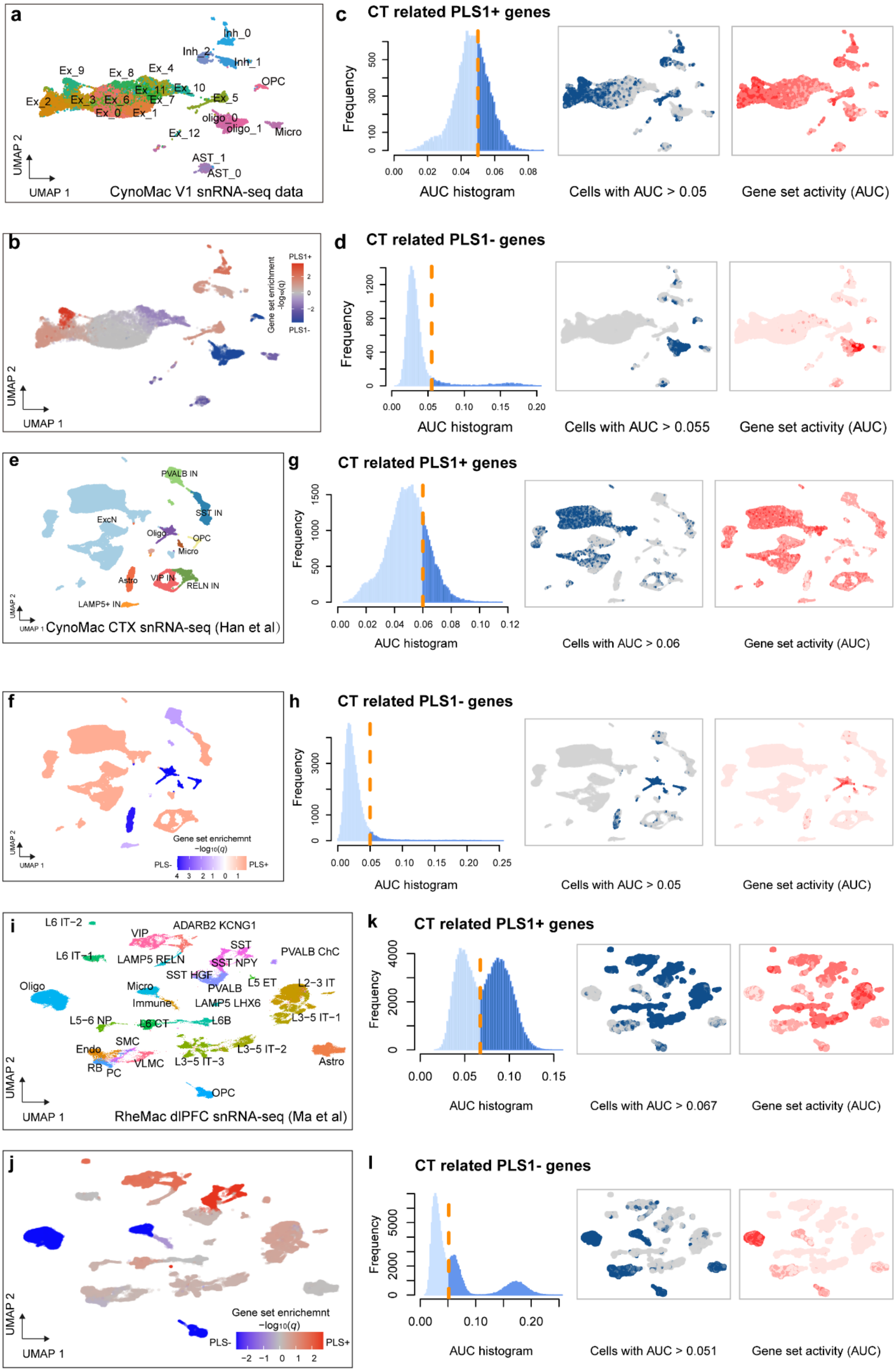

Supplementary Fig. 13 Cell cluster annotations and cell type enrichment of 1,005 CT-related genes annotated with three independent snRNA-seq datasets using AUCell. UMAP visualization of cell type clusters (**a, e, i**) and characterization of the enrichment of the CT-correlated

PLS1+ (red) and PLS1- (blue) genes with highly expressed marker genes (**b, f, j**) and AUCell analyses (**c-d, g-h, k-l**). Source data are provided as a Source Data file.

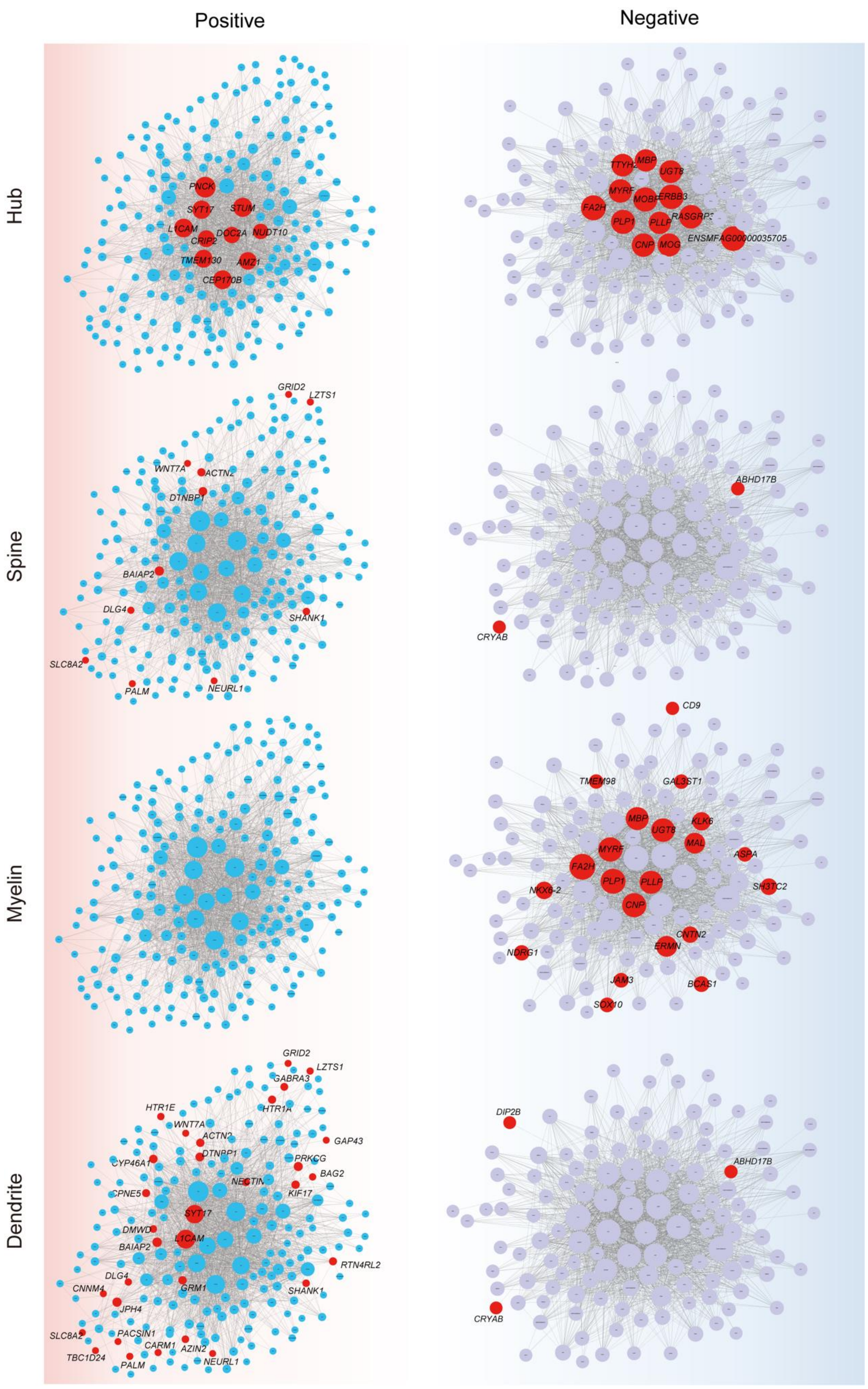

Positive
Negative
Hub
Spine
Myelin
Dendrite
PNCK
SYT17
STUM
L1CAM
CRIP2
DOC2A
NUDT10
TMEM130
AMZ1
CEP170B
TTYH2
MBP
UGT8
MYRF
MOBP
ERBB3
FA2H
PLP1
PLLP
RASGRP3
CNP
MOG
ENSMFAG00000035705
GRID2
LZTS1
WNT7A
ACTN2
DTNBP1
BAIAP2
DLG4
SHANK1
SLC8A2
PALM
NEURL1
ABHD17B
CRYAB
CD9
TMEM98
GAL3ST1
MBP
UGT8
KLK6
MAL
MYRF
ASPA
FA2H
PLP1
PLLP
SH3TC2
NKX6-2
CNP
CNTN2
ERMN
NDRG1
JAM3
BCAS1
SOX10
GRID2
LZTS1
GABRA3
HTR1E
HTR1A
WNT7A
ACTN2
GAP43
DTNBP1
CYP46A1
PRKCG
BAG2
NECTIN
CPNE5
KIF17
SYT17
DMWD
L1CAM
BAIAP2
RTN4RL2
DLG4
GRM1
SHANK1
CNNM4
JPH4
SLC8A2
PACSIN1
CARM1
AZIN2
TBC1D24
NEURL1
PALM
DIP2B
ABHD17B
CRYAB

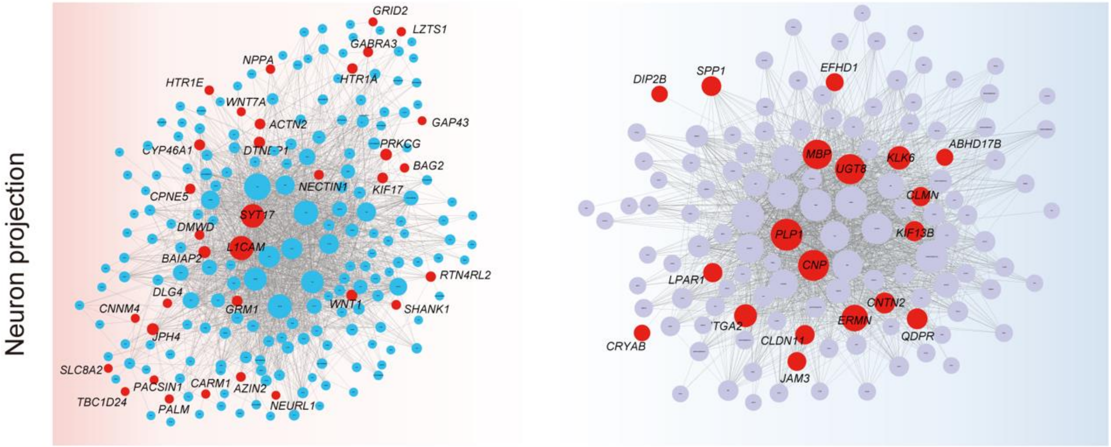

Supplementary Fig. 14 Visualization of the positive and negative weighted genes in 1,005 CT-related genes (weights > 0.1). Genes in the corresponding module are in red circle.

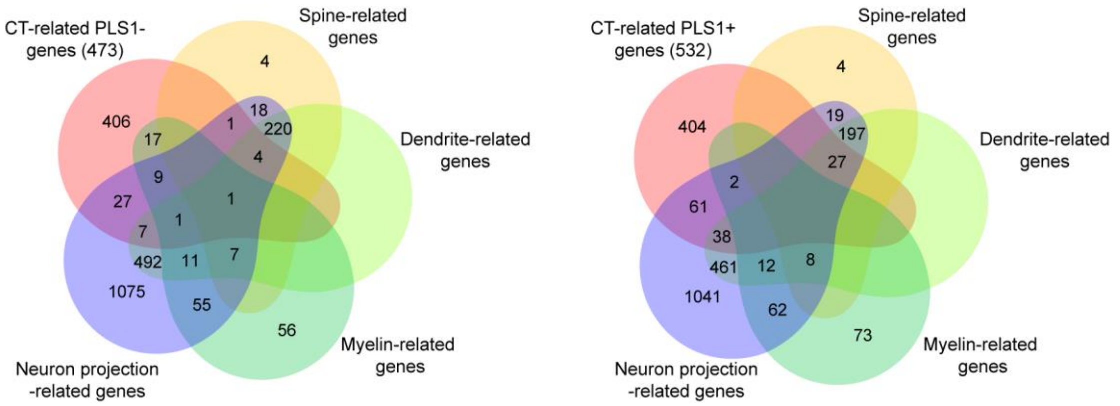

Supplementary Fig. 15 Overlapping of CT-related PLS1 genes and gene panels of spine, dendrite, myelin, and neuron projection.

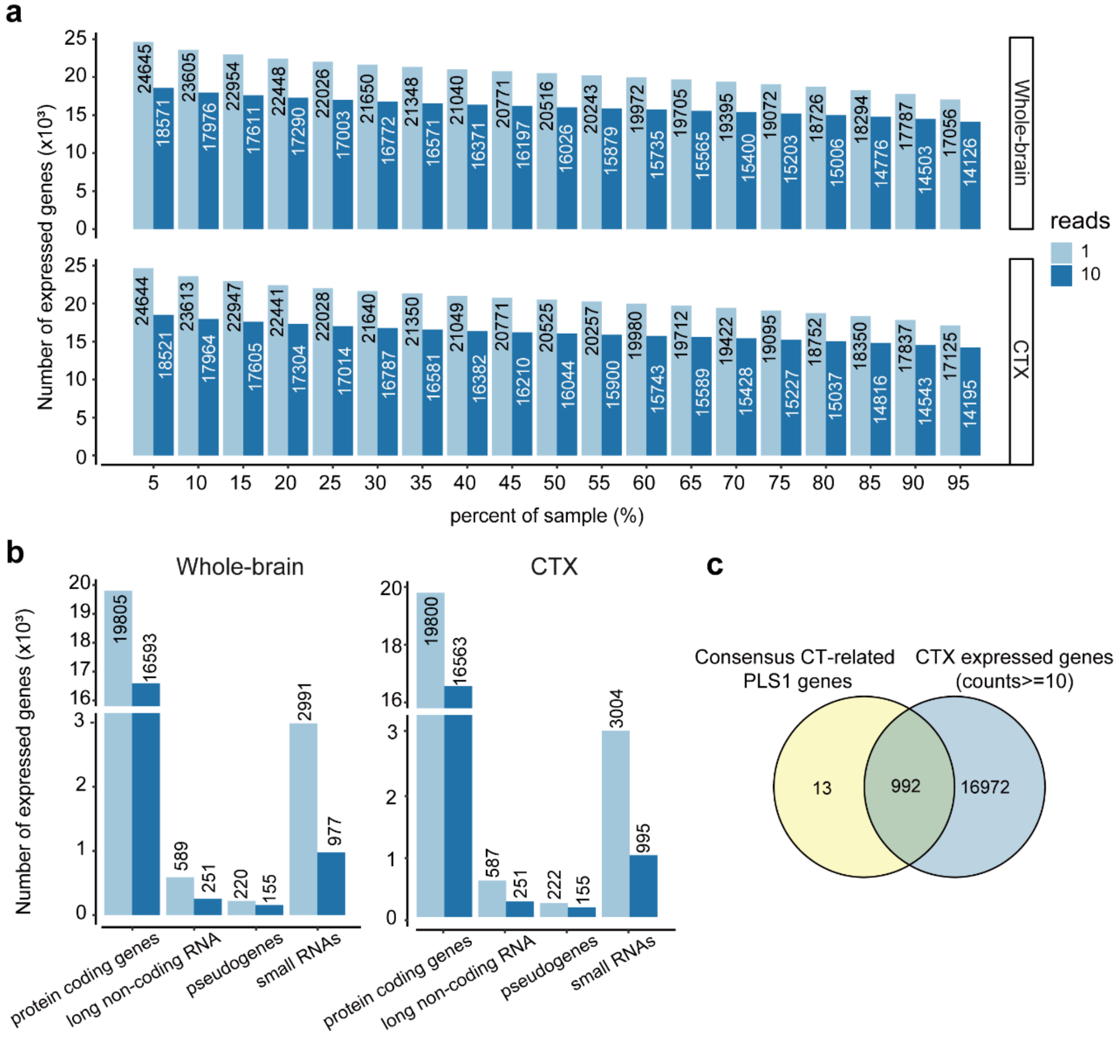

Supplementary Fig. 16 Genes retained across different combinations of minimum count and minimum number of samples. a Numbers of expressed genes retained with different minimum count (1 or 10) and samples across the whole-brain and cortical regions. **b** Number of compositions of expressed genes cut with read count of 1 and 10. **c** Overlapping of 1,005 consensus CT-related PLS1 genes and the total cortical expressed genes with read count ≥ 10.

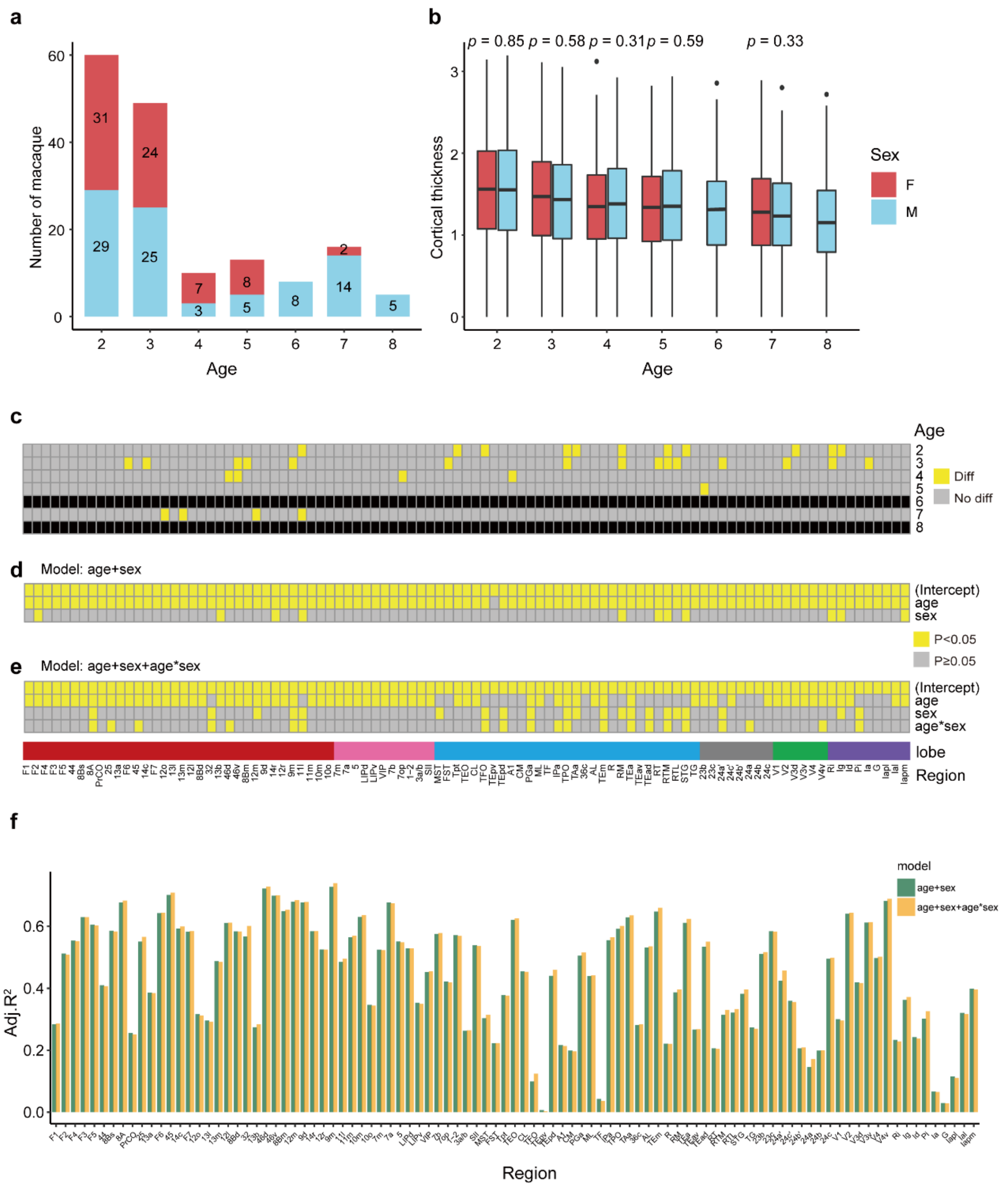

Supplementary Fig. 17 Linear modelling the effect of sex and age. a Distribution of individuals by age and sex. **b** No significant group difference of cortical thickness across age 2 to 8 and female subjects were missing at age 6 and 8. The numbers of subjects used for comparison at individual age were shown in **a**. In each box plot, the center line indicates the median, the edges of the box indicate the 25th and 75th percentile (interquartile range, IQR) and the whiskers indicate last point within a 1.5x IQR (n = 97 regions). *p*-values are determined by two-sided Wilcoxon rank sum test and uncorrected for multiple comparisons. **c** Region-level group difference of cortical thickness among male and female subjects across age 2 to 8. Statistical differences between the groups are determined by two-sided Wilcoxon rank sum test. The numbers of subjects used for comparison at individual age are also shown in **a**. Yellow boxes represent significant sex difference (FDR corrected $p < 0.05$). **d** Region-level significance of the intercept of linear model of age and sex. **e** Region-level significance of the intercept of linear model of age, sex and age*sex. **f** Comparison of adjust R^2 of

the models with or without age*sex. Source data are provided as a Source Data file.

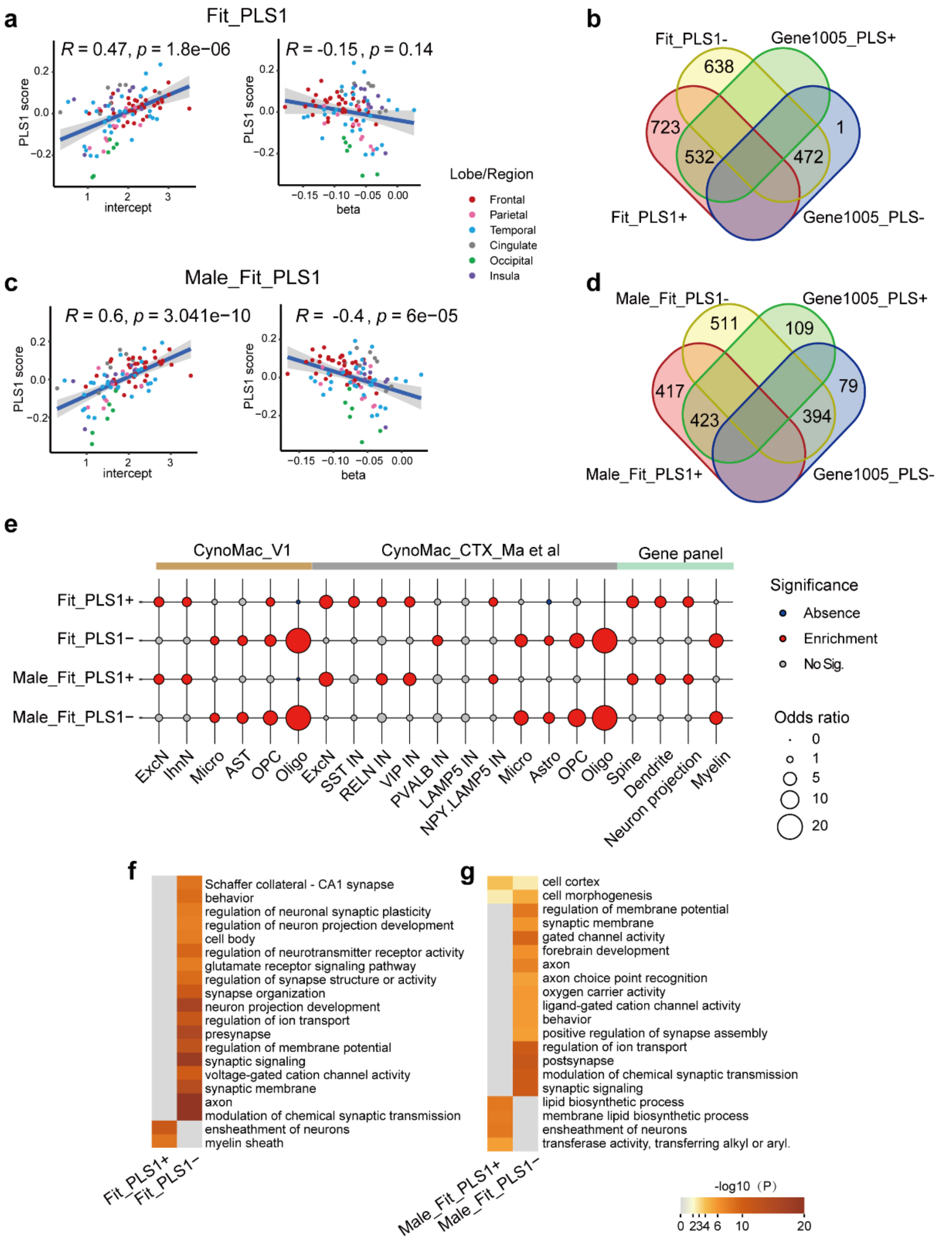

Supplementary Fig. 18 Linear modelling of age and evaluation the results in male-only data. **a** A linear model considering only the effect of age to derive its beta coefficient and intercept as inputs to PLS model. **b** Overlapping of 1,005 consensus CT-related genes and 2,365 genes identified using both male and female data. **c** A linear model considering only the effect of age and PLS model with **male-only** imaging and transcriptomics data. **d** Overlapping of 1,005 consensus CT-related genes and 1,745 genes identified in male-only data. **e** Enrichment of cell types with snRNA-seq data obtained from V1, from previously published data [5] and four gene panels. **f, g** Functional enrichment of GO biological functions and KEGG pathways was performed using Metascape [7] and top 20 significant GO terms/pathways ($p < 0.05$, one-sided hypergeometric test, uncorrected) are shown.

The blue line in **a**, **c** is the fitted line between PLS1 score and beta coefficient/intercept and grey shaded area indicates the 95% confidence intervals. *p*-values in **a**, **c** are determined based on a two-sided test and not corrected for multiple comparisons. Each point stands for a cortical region (n = 97 regions). Source data are provided as a Source Data file.

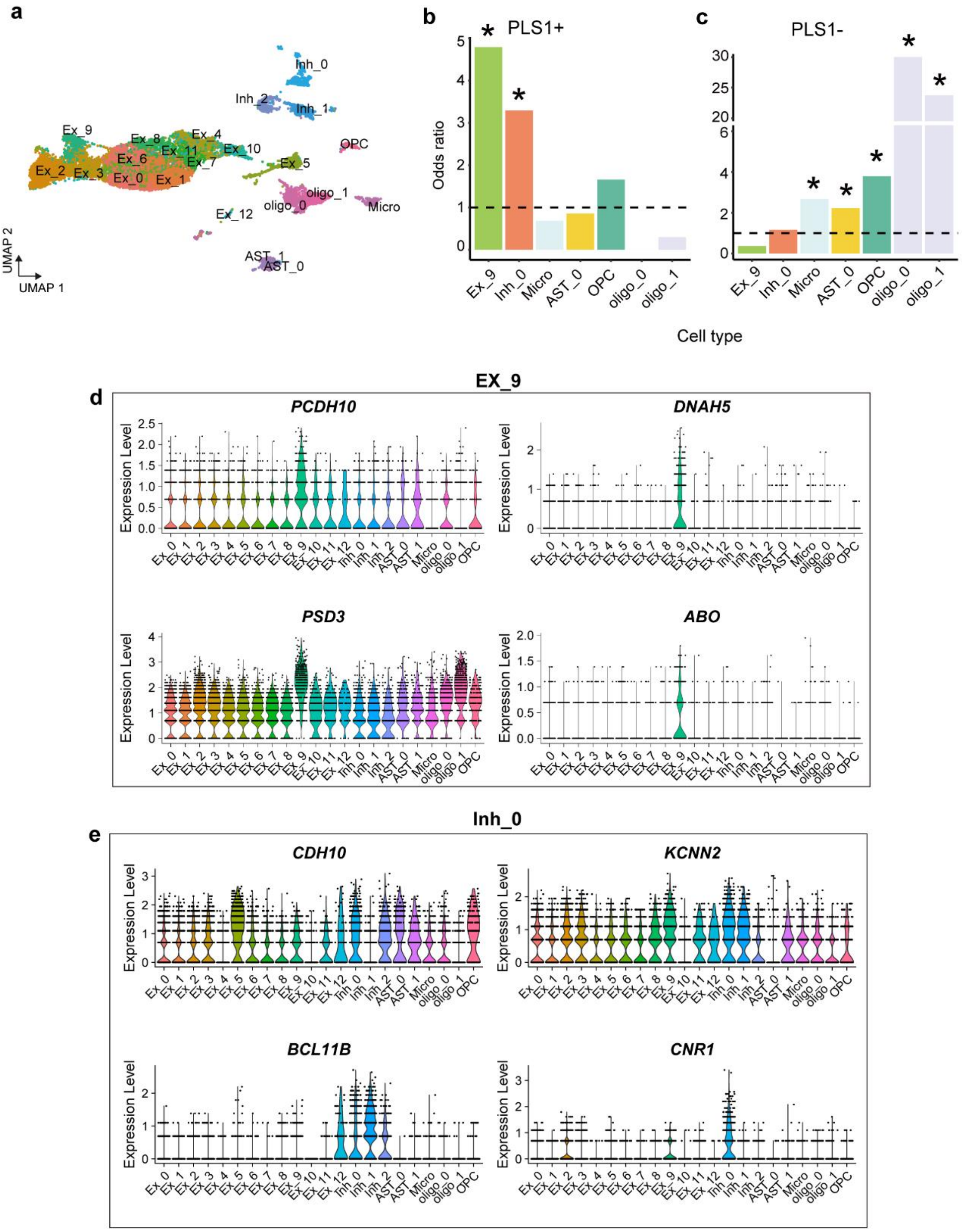

Supplementary Fig. 19 Cell subtype enrichment for 1,005 CT-related genes based on snRNA-seq data obtained from V1 and examples of cell type-specific genes. a UMAP visualization of different cell subtype clusters. The classification of single cells with each color represents a pre-defined cell type. **b-c** Enriched cell types in CT-correlated PLS1+ and PLS1- genes respectively. *p*-values are determined based on two-sided Fisher's exact test and the asterisks denote odds ratio > 1 (y-axis) and FDR corrected $p < 0.05$. **d** Example genes in Ex_9 subtype of excitatory neurons. **e** Example genes in Inh_0 subtype of inhibitory neurons. Source data are provided as a Source Data file.

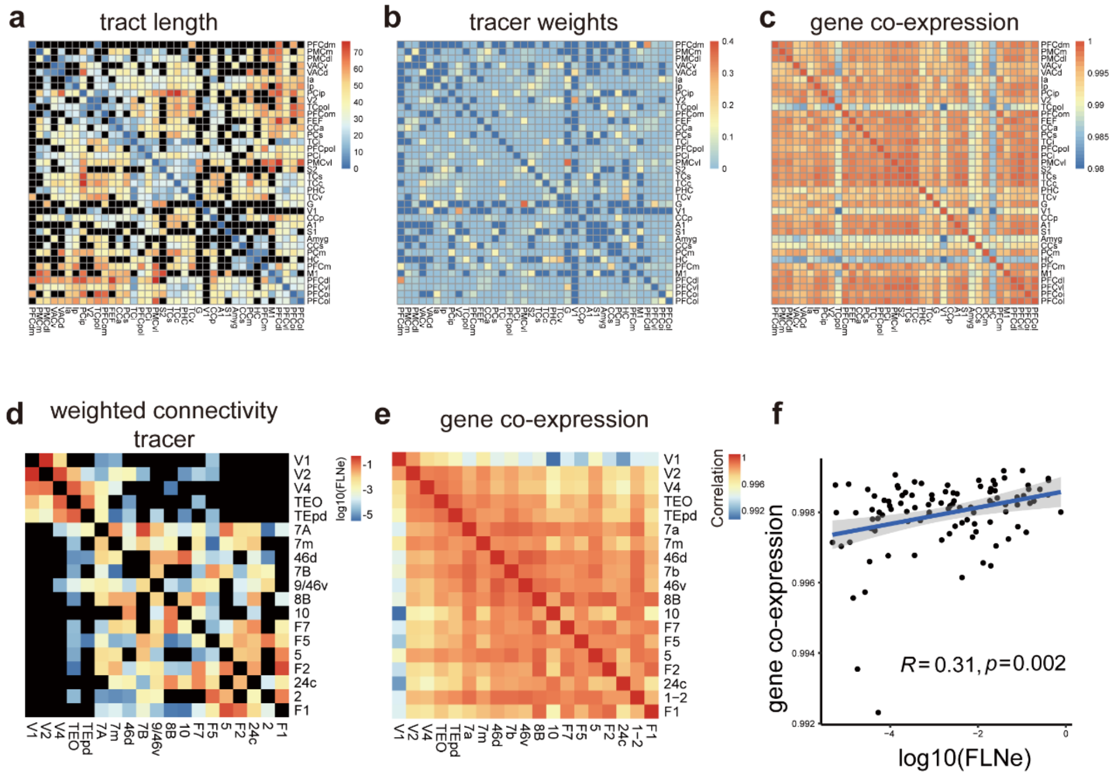

Supplementary Fig. 20 Correspondence between tracer-, tractography-derived structural connectivity matrices and regional gene co-expression. Individual tractography- (**a**) and tracer- (**b**) derived weighted connectivity matrices [8] and gene co-expression correlation matrix (**c**) based on the RM parcellation. Tracer- (**d**) [9] and gene co-expression matrices (**e**) of 19 ROIs are significantly correlated (R = 0.31, p = 0.002) (**f**). The blue line in **f** is the fitted line between tracer connectivity and gene co-expression and grey shaded area indicates the 95% confidence intervals. p-value is determined based on a two-sided test and not corrected for multiple comparisons. Each point stands for a region pair (n = 101). Source data are provided as a Source Data file.

References:

1. Richiardi J*, et al.* BRAIN NETWORKS. Correlated gene expression supports synchronous activity in brain networks. *Science* **348**, 1241-1244 (2015).

2. Hawrylycz M*, et al.* Canonical genetic signatures of the adult human brain. *Nat Neurosci* **18**, 1832-1844 (2015).

3. Hawrylycz MJ*, et al.* An anatomically comprehensive atlas of the adult human brain transcriptome. *Nature* **489**, 391-399 (2012).

4. Hansen JY*, et al.* Mapping neurotransmitter systems to the structural and functional organization of the human neocortex. *Nat Neurosci* **25**, 1569-1581 (2022).

5. Han L*, et al.* Cell transcriptomic atlas of the non-human primate Macaca fascicularis. *Nature* **604**, 723-731 (2022).

6. Ma S*, et al.* Molecular and cellular evolution of the primate dorsolateral prefrontal cortex. *Science* **377**, eabo7257 (2022).

7. Zhou Y*, et al.* Metascape provides a biologist-oriented resource for the analysis of systems-level datasets. *Nat Commun* **10**, 1523 (2019).

8. Shen K, Bezgin G, Schirner M, Ritter P, Everling S, McIntosh AR. A macaque connectome for large-scale network simulations in TheVirtualBrain. *Sci Data* **6**, 123 (2019).

9. Markov NT*, et al.* A weighted and directed interareal connectivity matrix for macaque cerebral cortex. *Cereb Cortex* **24**, 17-36 (2014).
