# Supplementary material for: Brain-wide and cell-specific transcriptomic insights into MRI-derived cortical morphology in macaque monkeys
Source: Nat Commun. 2023 Mar 17;14:1499. doi: 10.1038/s41467-023-37246-w (PMC10023667; doi:10.1038/s41467-023-37246-w)
Supplement: Supplementary file 2 — Description of Additional Supplementary Files [file 41467_2023_37246_MOESM2_ESM.pdf]

Description of Additional Supplementary Files

File Name: Supplementary Data 1
Description: Information of sequenced samples

File Name: Supplementary Data 2
Description: Expressed genes aligned to mfas5

File Name: Supplementary Data 3
Description: Cortical expressed gene list used in WGCNA

File Name: Supplementary Data 4
Description: KEGG pathway enrichment for M1-M4

File Name: Supplementary Data 5
Description: Neurotransmitter enrichment in M1-M4

File Name: Supplementary Data 6
Description: Co-expression clustering of transmitter related genes

File Name: Supplementary Data 7
Description: Information of module view for M1, M2 and M4

File Name: Supplementary Data 8
Description: Cell type enrichment in 20 modules and M1-M4

File Name: Supplementary Data 9
Description: Explained variances of PLS1 in age 2 to age 8

File Name: Supplementary Data 10
Description: Significant CT-correlated gene list (1,284) across age 2 to age 8

File Name: Supplementary Data 11
Description: 1,005 CT-related gene list

File Name: Supplementary Data 12
Description: Overlap of 1,005 CT-related genes with available CT-related genes in humans

File Name: Supplementary Data 13
Description: Cell type enrichment in CT-related genes

File Name: Supplementary Data 14
Description: Biological processes and KEGG pathways enrichment for CT-related genes

File Name: Supplementary Data 15
Description: Overlap of CT-related genes and 4 gene panels
