# Supplementary material for: Brain-wide and cell-specific transcriptomic insights into MRI-derived cortical morphology in macaque monkeys
Source: Nat Commun. 2023 Mar 17;14:1499. doi: 10.1038/s41467-023-37246-w (PMC10023667; doi:10.1038/s41467-023-37246-w)
Supplement: Supplementary file 3 — Supplementary Data 1-15 [file 41467_2023_37246_MOESM3_ESM.zip › SupplementaryData/Supplementary Data 6 Co-expression clustering of transmitter related genes.docx]

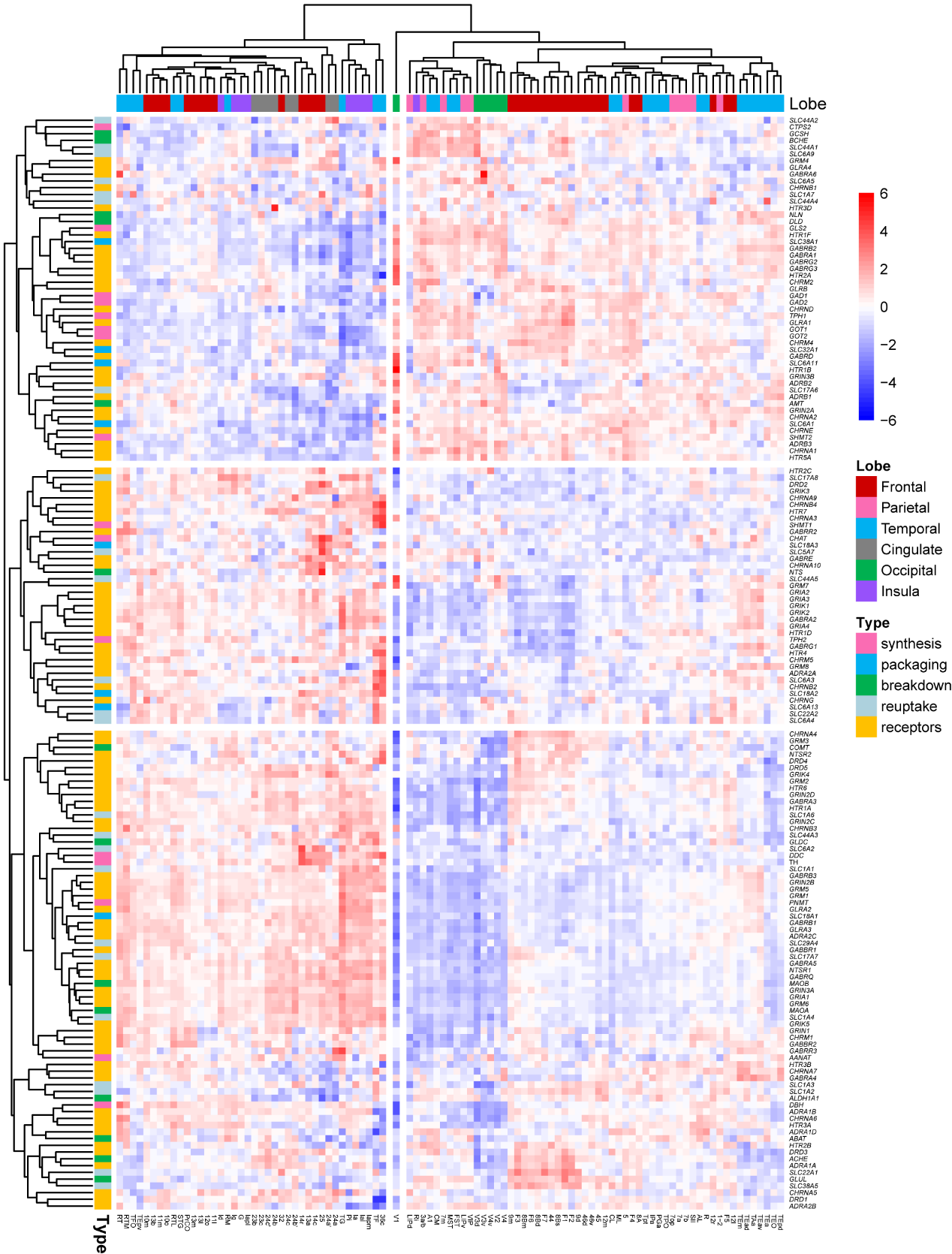


**Supplementary Data 6-1** Heatmaps of regional variation of 8 different neurotransmitters and their receptors (160 genes) across the entire neocortex.


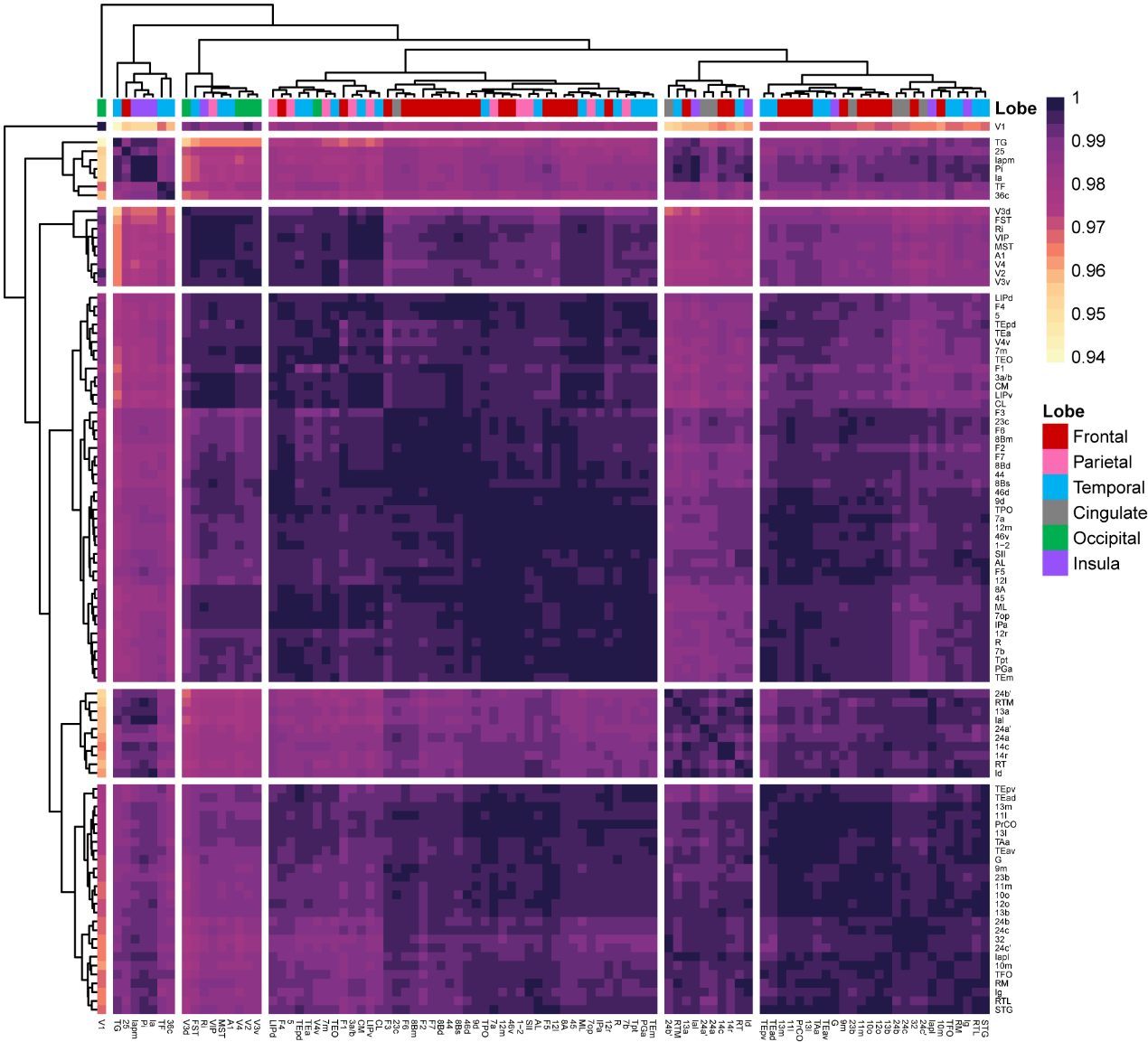


**Supplementary Data 6-2** Heatmaps showing pairwised Spearman correlation between the cortical subregions based on expression profiles of 8 different neurotransmitters and their receptors.


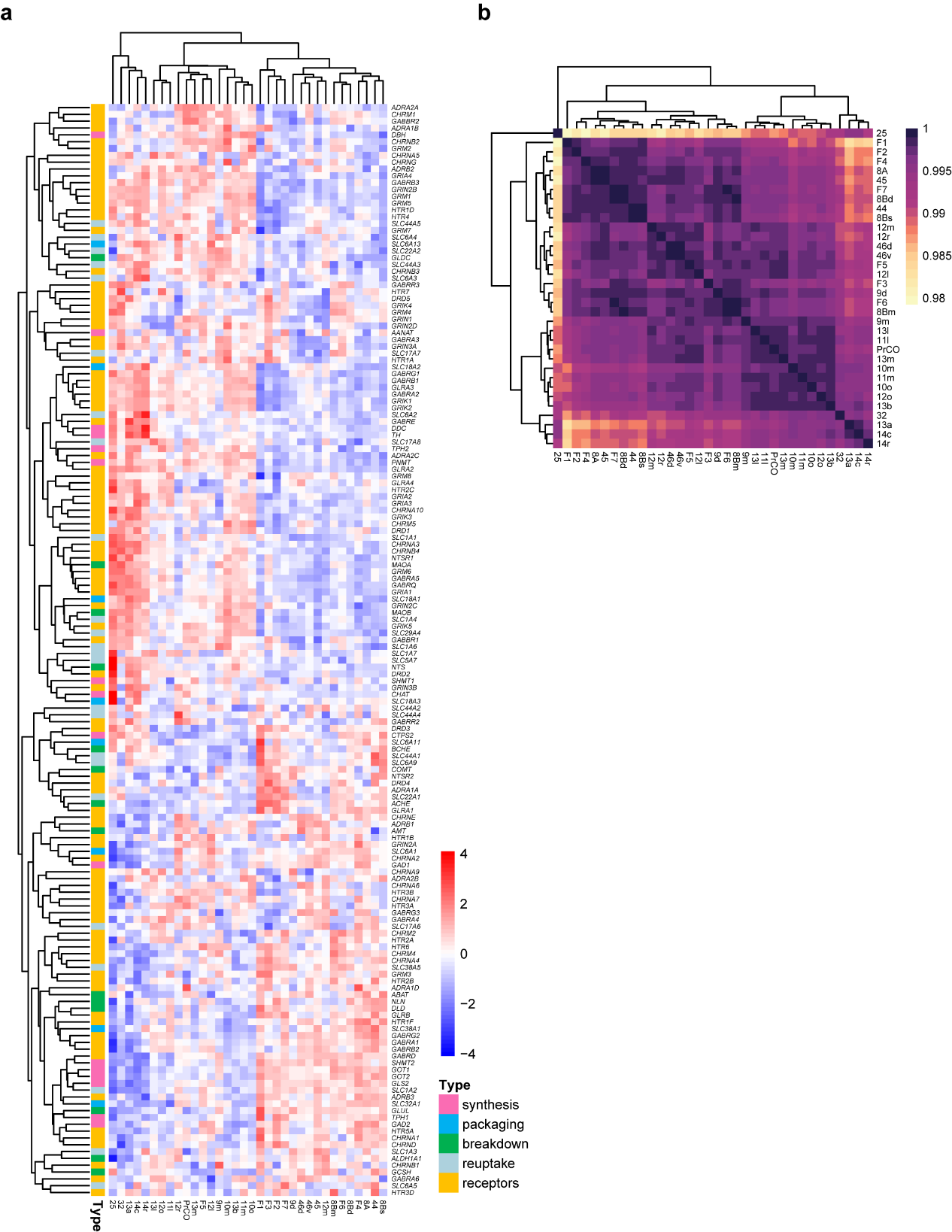


**Supplementary Data 6-3** Heatmaps showing dorsolateral-ventromedial variation in **frontal lobe** based on the gene expression of 8 neurotransmitters and their receptors.


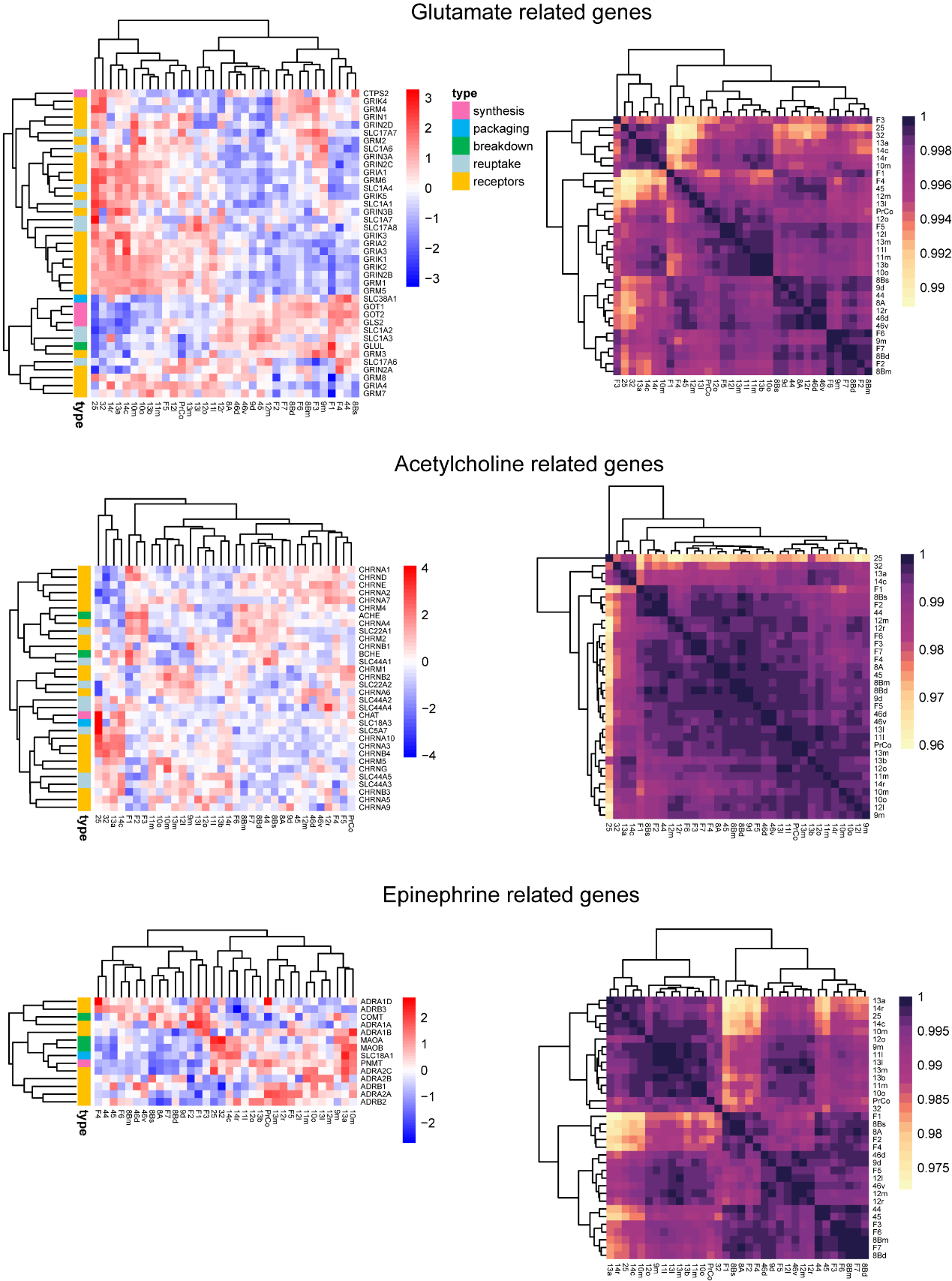


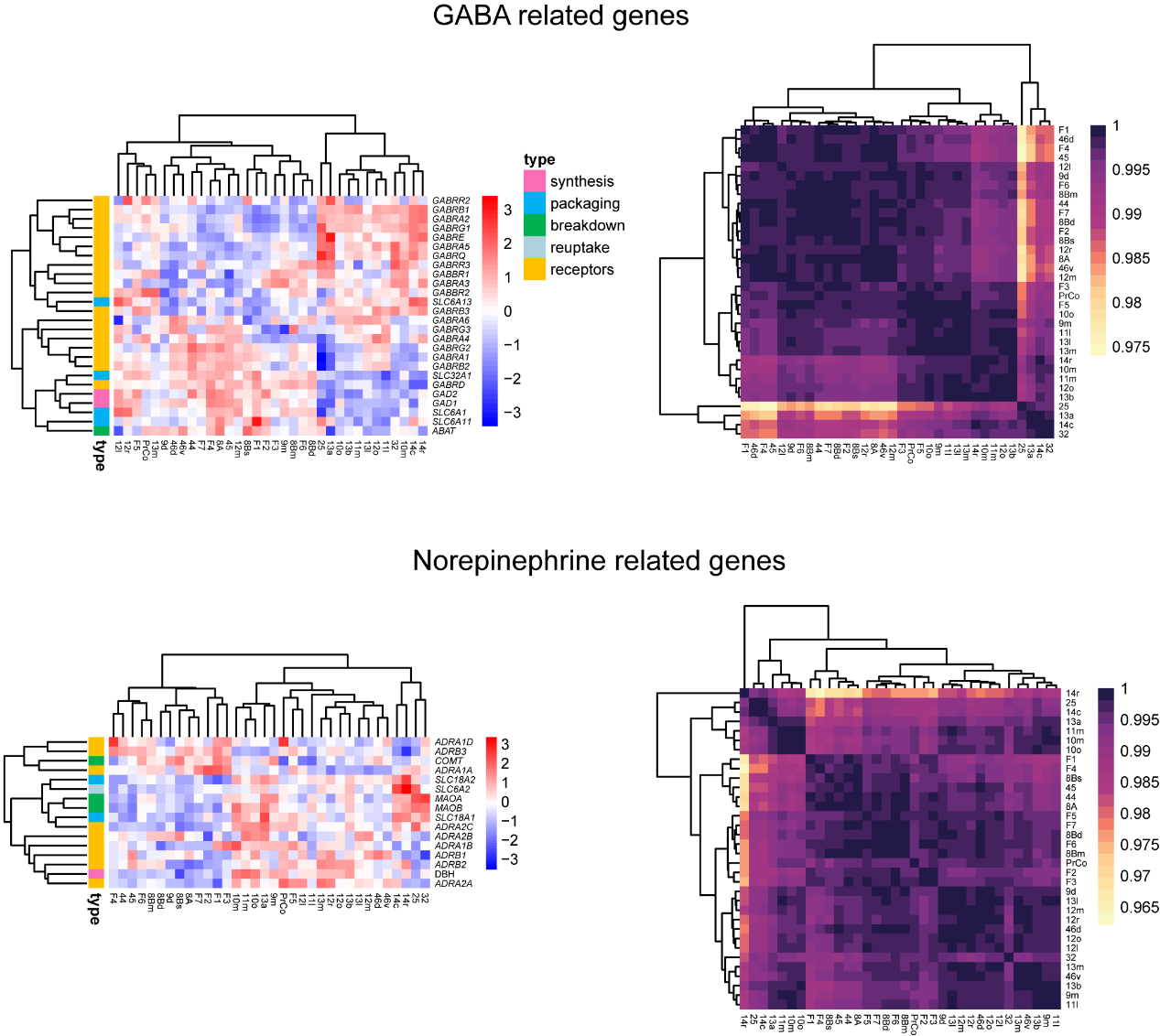


**Supplementary Data 6-3** Heatmaps showing similar distinct dorsolateral-ventromedial pairwised Spearman correlation variation in frontal lobe based on expression profiles of glutamate, acetylcholine, epinephrine, GABA, norepinephrine.
