# Supplementary material for: Brain-wide and cell-specific transcriptomic insights into MRI-derived cortical morphology in macaque monkeys
Source: Nat Commun. 2023 Mar 17;14:1499. doi: 10.1038/s41467-023-37246-w (PMC10023667; doi:10.1038/s41467-023-37246-w)
Supplement: Supplementary file 4 — Reporting Summary [file 41467_2023_37246_MOESM4_ESM.pdf]

nature portfolio

Corresponding author(s): Zheng Wang；Guang-Zhong Wang；Meiyun Wang

Last updated by author(s): Feb 15, 2023

Reporting Summary

Nature Portfolio wishes to improve the reproducibility of the work that we publish. This form provides structure for consistency and transparency in reporting. For further information on Nature Portfolio policies, see our Editorial Policies and the Editorial Policy Checklist.

Statistics

For all statistical analyses, confirm that the following items are present in the figure legend, table legend, main text, or Methods section.

n/a	Confirmed	
☐	☒	The exact sample size (*n*) for each experimental group/condition, given as a discrete number and unit of measurement
☐	☒	A statement on whether measurements were taken from distinct samples or whether the same sample was measured repeatedly
☐	☒	The statistical test(s) used AND whether they are one- or two-sided<br>*Only common tests should be described solely by name; describe more complex techniques in the Methods section.*
☐	☒	A description of all covariates tested
☐	☒	A description of any assumptions or corrections, such as tests of normality and adjustment for multiple comparisons
☐	☒	A full description of the statistical parameters including central tendency (e.g. means) or other basic estimates (e.g. regression coefficient) AND variation (e.g. standard deviation) or associated estimates of uncertainty (e.g. confidence intervals)
☐	☒	For null hypothesis testing, the test statistic (e.g. *F*, *t*, *r*) with confidence intervals, effect sizes, degrees of freedom and *P* value noted<br>*Give P values as exact values whenever suitable.*
☒	☐	For Bayesian analysis, information on the choice of priors and Markov chain Monte Carlo settings
☒	☐	For hierarchical and complex designs, identification of the appropriate level for tests and full reporting of outcomes
☐	☒	Estimates of effect sizes (e.g. Cohen's *d*, Pearson's *r*), indicating how they were calculated

Our web collection on statistics for biologists contains articles on many of the points above.

Software and code

Policy information about availability of computer code

Data collection	The raw fastq files of RNA-seq were obtained from Illumina platform and the raw sequence data of single nucleus RNA-seq were obtained from 10X genomics. MRI dataset were collected on 3.0 T MRI scanners. (See Methods)
Data analysis	-Trim Galore(v0.6.0), https://www.bioinformatics.babraham.ac.uk/projects/trim_galore/<br>-FASTX-Toolkit(v 0.0.14),http://hannonlab.cshl.edu/fastx_toolkit/<br>-STAR(v2.7.3a) , https://github.com/alexdobin/STAR<br>-featureCounts(v2.0.1),http://subread.sourceforge.net/<br>-RSEM(v1.2.28) ,http://deweylab.github.io/RSEM/<br>-Cellranger toolkit (v6.1.1) ,https://support.10xgenomics.com/single-cell-gene-expression/software/overview/welcome<br>-Seurat package(v4.1.0) ,https://satijalab.org/seurat/<br>-R(v4.1.0), https://www.R-project.org/<br>-MATLAB(2012a-2017a)<br>-Picard Toolkit (v2.21.2), http://broadinstitute.github.io/picard/<br>-Subread_to_DEXSeq, https://github.com/vivekbhr/Subread_to_DEXSeq<br>-Metascape, https://www.metascape.org/<br>-FSL(v6.0.3,https://fsl.fmrib.ox.ac.uk/fsl/)<br>-CMTK (v3.3.1),http://nitrc.org/projects/cmtk/<br>-ANTs (v3.14.5), http://stnava.github.io/ANTs/<br>-AFNI (v20.1.13), https://afni.nimh.nih.gov/

-DESeq2 (v1.36.0), https://bioconductor.org/packages/release/bioc/html/DESeq2.html
-edgeR (v3.38.4), https://bioconductor.org/packages/release/bioc/html/edgeR.html
-limma (v3.52.4), https://bioconductor.org/packages/release/bioc/html/limma.html
-WGCNA (v1.70), https://horvath.genetics.ucla.edu/html/CoexpressionNetwork/Rpackages/WGCNA/
-Custom codes used in the analysis can be accessed using the github link https://github.com/WangLab-SINH/Macaque_Brain_Transcriptome_MRI.

For manuscripts utilizing custom algorithms or software that are central to the research but not yet described in published literature, software must be made available to editors and reviewers. We strongly encourage code deposition in a community repository (e.g. GitHub). See the Nature Portfolio guidelines for submitting code & software for further information.

Data

Policy information about availability of data

All manuscripts must include a data availability statement. This statement should provide the following information, where applicable:

- Accession codes, unique identifiers, or web links for publicly available datasets
- A description of any restrictions on data availability
- For clinical datasets or third party data, please ensure that the statement adheres to our policy

The RNA-seq data generated in this study have been deposited in the Sequence Read Archive (SRA) under accession code PRJNA905082 (https://www.ncbi.nlm.nih.gov/bioproject/PRJNA905082).
The public datasets used in this study can be accessed as described below:
The single-cell RNA-seq data of macaque neocortex is available at https://db.cngb.org/nhpca/.
The snRNA-seq data of the macaque dlPFC is available at http://resources.sestanlab.org/PFC/.
The cynomolgus macaque genome Ensembl Macaca_fascicularis_5.0 is available at https://www.ensembl.org.
D99 template of macaque brain is available at https://afni.nimh.nih.gov/pub/dist/atlases/macaque/.
Subcortical atlas of macaque can be downloaded from https://afni.nimh.nih.gov/pub/dist/doc/htmldoc/nonhuman/macaque_tempatl/atlas_sarm.html.
The Cynomolgus macaque template (Cyno162) is available at https://doi.org/10.1093/cercor/bhaa229.
Allen Human Brain Atlas data is available at https://human.brain-map.org/.
Predicted comprehensive human mRNA expression data is available at http://www.meduniwien.ac.at/neuroimaging/mRNA.html.
Results and statistics related to main figures were provided in Supplementary Data 1-15.
Source data are provided with this paper.

Human research participants

Policy information about studies involving human research participants and Sex and Gender in Research.

Reporting on sex and gender	NA
Population characteristics	NA
Recruitment	NA
Ethics oversight	NA

Note that full information on the approval of the study protocol must also be provided in the manuscript.

Field-specific reporting

Please select the one below that is the best fit for your research. If you are not sure, read the appropriate sections before making your selection.

[x] Life sciences   [ ] Behavioural & social sciences   [ ] Ecological, evolutionary & environmental sciences

For a reference copy of the document with all sections, see nature.com/documents/nr-reporting-summary-flat.pdf

Life sciences study design

All studies must disclose on these points even when the disclosure is negative.

Sample size	Sample size was not pre-determined.Totally, 878 bulk tissue samples and 17509 single nuclei were sequenced. The sample size allowed us to obtain high coverage transcriptome of brain regions, and perform downstream analysis. The structure MRI dataset included 162 cynomolgus macaques. The sample size allowed us to obtain an estimate of cortical thickness at different ages.

Data exclusions	For bulk RNA-seq analysis, samples with low quality (% unique mapping < 50%, Z > 2 for %Intergenic Bases, GC Dropout rate, or AT Dropout rate and Z < − 2 for %High-quality Aligned Reads, %mRNA Bases, or Median 5′ to 3′ Bias) were identified as outliers, and any sample with greater than two outlier indexes was removed. Totally, 59 samples were removed. For snRNA-seq analysis, we excluded nuclei based on quality control procedure and nuclei with detected genes less than 500 and more than 7500 were removed for further analysis. One subject at age 9 was excluded for MRI data analysis as only one subject at that age.
Replication	Two snRNA-seq data in macaques were analyzed to ensure reproducibility.<br>Cell type and functional enrichments for WGCNA modules and 1,005 CT-related genes were replicated with these two snRNA-seq data.<br>AUCell and marker genes sensitivity analyses were also applied.<br>Different combinations of minimum count and minimum number of samples were test for evaluation the expressed genes and CT-related genes retained.
Randomization	All group assignments were pre-determined based on known age. To robustly estimate p values in our neuroimagingtranscriptomic analyses, we employ multiple randomization (permutation) strategies. To examine the effect of sex on our results, we replicated the above analyses using male-only imaging and transcriptome data. See Methods for additional details on these procedures.
Blinding	Blinding was not relevant for the study. All group assignments were known based on known ages. Distribution of individuals in MRI data by age and sex was provided (Supplementary Fig. 17).

Reporting for specific materials, systems and methods

We require information from authors about some types of materials, experimental systems and methods used in many studies. Here, indicate whether each material, system or method listed is relevant to your study. If you are not sure if a list item applies to your research, read the appropriate section before selecting a response.

Materials & experimental systems

n/a	Involved in the study
☐	☒ Antibodies
☒	☐ Eukaryotic cell lines
☒	☐ Palaeontology and archaeology
☐	☒ Animals and other organisms
☒	☐ Clinical data
☒	☐ Dual use research of concern

Methods

n/a	Involved in the study
☒	☐ ChIP-seq
☒	☐ Flow cytometry
☐	☒ MRI-based neuroimaging

Antibodies

Antibodies used	Mouse anti-NeuN, clone A60, Alexa Fluor 555 conjugate, Cat.# MAB377A5 [Lot # 3299928]
Validation	The validation by manufactures: https://www.merckmillipore.com/CN/zh/product/Anti-NeuN-Antibody-clone-A60-Alexa-Fluor-555-Conjugate,MM_NF-MAB377A5.

Animals and other research organisms

Policy information about studies involving animals; ARRIVE guidelines recommended for reporting animal research, and Sex and Gender in Research

Laboratory animals	For transcriptomics data, ages of each adult monkeys (Macaca fascicularis) were provided in Supplementary Data 1.<br>For MRI data, 161 Macaca fascicularis ranging between 2 and 8 years (only one subject at age 9 was excluded hence) was included. Distribution of individuals by age was provided in Supplementary Fig. 17.
Wild animals	This study did not involve wild animals
Reporting on sex	For transcriptomics data, 8 male and 1 female monkeys were included. Similar expression pattern was observed between male and feamle macaques (r=0.964) and PCA analysis revealed that sex is not the major source of variation. For MRI data, 72 female subjects and 89 male subjects were involved. The effect of the sex on the PLS analysis was also evaluated using linear modelling and male-only imaging and transcription data.
Field-collected samples	This study did not involve samples collected from the field.
Ethics oversight	All animal experimental procedures were approved by the Animal Care and Use Committee of CAS Center for Excellence in Brain Science and Intelligence Technology, Chinese Academy of Sciences.

Note that full information on the approval of the study protocol must also be provided in the manuscript.

Magnetic resonance imaging

Experimental design

Design type	Structural MRI
Design specifications	Five to 7 sets of whole-brain images for each animal. Detailed information was provided in the published paper (doi: 10.1093/cercor/bhaa229).
Behavioral performance measures	Only structural MRI was used in the present study and no behavioral performance was measured.

Acquisition

Imaging type(s)	Structural
Field strength	3 Tesla
Sequence & imaging parameters	High-resolution T1-weighted anatomical images of macaque brain were acquired with key parameters as follows: TR = 2300 ms; TE = 3 ms; inversion time = 1000 ms; flip angle = 9◦; acquisition voxel size = 0.5 × 0.5 × 0.5 mm3. Five to 7 whole-brain anatomical volumes were recorded for each subject. See Method and published paper of the MRI datasets (doi: 10.1093/cercor/bhaa229)
Area of acquisition	Whole brain scan

Diffusion MRI ☐ Used ☒ Not used

Preprocessing

Preprocessing software	-Motion correction: FMRIB's Linear Image Registration Tool in FMRIB Software Library (FSL) (http://www.fmrib.ox.ac.uk/fsl/)<br>-Intensity bias correction:CMTK's (http://nitrc.org/projects/cmtk/) mrbias tool<br>-Symmetric group-wise normalization template building algorithm provided by ANTs software (http://stnava.github.io/ANTs/)<br>-Skull-stripped combining automated (FSL Brain Extraction Tool [BET])<br>-Segmentation (FMRIB's Automated Segmentation Tool within FSL)<br>-Cortical thickness estimated using the diffeomorphic registration-based cortical thickness (DiReCT) method implemented in ANTs
Normalization	Individual brain was normalized to a brain atlas of cynomolgus macaques using ANTs. Detailed information was provided in the published paper (doi: 10.1093/cercor/bhaa229).
Normalization template	D99 template
Noise and artifact removal	Detailed information was provided in the published paper (doi: 10.1093/cercor/bhaa229).
Volume censoring	Data was not volume censored.

Statistical modeling & inference

Model type and settings	For age trajectory of normative structural variation, Gaussian process regression was developed and used age as a covariate to predict brain measures.Detailed information was available in the published paper (doi: 10.1093/cercor/bhaa229).<br>For imaging-transcriptomics analysis, multivariate methods (PLS regression) was performed. The statistical significance of the PLS explained variance was evaluated by permuting the response variables 10000 times.
Effect(s) tested	See previous point.

Specify type of analysis: ☒ Whole brain ☐ ROI-based ☐ Both

Statistic type for inference (See Eklund et al. 2016)	Whole-brain parcellation continuous values were carried forward to neuroimaging-transcriptomic analyses.
Correction	See Methods and above for descriptions on the multiple permutation strategies employed to test robustness of empirical effects.

Models & analysis

n/a	Involved in the study
☒	☐ Functional and/or effective connectivity
☒	☐ Graph analysis
☐	☒ Multivariate modeling or predictive analysis

Multivariate modeling and predictive analysis	Partial least squares regression was used relate the regional cortical thickness to 23,613 cortical genes, and to rank genes based on their spatial relationship to brain cortical thickness maps. This procedure was repeated for different ages from 2 to 8, and genes that exhibited persistent associations with CT across all age groups were assembled as consensus PLS1 genes.
